# Identification, Physiological Time and Reproductive Potential of *Bradysia impatiens* (Diptera: Sciaridae) from Liuba, Shaanxi, with a Review Pertaining to Its Occurrences in China

**DOI:** 10.3390/biology15191696

**Published:** 2026-09-23

**Authors:** Qi Wang, Limin Zhao, Ya Sun

**Affiliations:** 1 School of Biological Science and Engineering, Shaanxi University of Technology, Hanzhong 723000, China; wangqis6@163.com (Q.W.); 18391507228@163.com (Y.S.); 2 Shaanxi Province Key Laboratory of Bioresources, Hanzhong 723000, China

**Keywords:** *Bradysia impatiens*, DNA barcoding, fecundity, hypopygium, identity, microphotograph, reciprocal model, thermal constant, threshold temperature, venation

## Abstract

The sciarid fly *Bradysia impatiens* (Johannsen) is one of the key pests on American ginseng *Panax quinquefolium* L. in Liuba County, Shaanxi Province, China. Its living specimens were sampled, isolated, reared, mounted, observed and microphotographed. Its identification was completed through both comparisons of morphotaxonomic characteristics and DNA barcoding, and its occurrences in China were reviewed. After conducting serial rearing experiments of the insect at eight temperatures, we calculated developmental threshold temperatures (*C*) and thermal constants (*K*) of the species in its egg, larval, pupal and generational stages, and then constructed four reciprocal models *N* = *K*/(*T* − *C*), where *N* denotes developmental duration and *T* is rearing temperature. With values of *K* and *C* as well as their *SE* written, the reciprocal model for its generational stage is *N_generation_* = (374.9 ± 8.15)/[*T* − (6.6 ± 0.29)]. Based on *C* and *K* and annual effective accumulated temperatures, we estimated that the sciarid pest would reproduce at a rate of 4.2 to 5.7 generations a year in local regions. In the laboratory, the fecundity of the species was tested to be 59.2 eggs per female, the survival rate of the immature individuals averaged 46.4% in the range from 10.0 to 30.0 °C, and the sex ratio of female to male was 2:1. Based on these coefficients, we are able to estimate the reproductive potential that a parent female and her offspring may reach in a year if they live under favorable conditions, and corresponding economic losses.

## Simple Summary

One of the sciarids, *Bradysia impatiens* (Johannsen), has attacked American ginseng in Liuba, Shaanxi, China, and caused severe damage. The species was first placed in the genus *Sciara*, with several synonyms, and moved formally to its present genus in 1966. By comparing characteristics and microphotographs with specific descriptions and drawings published, our morphotaxonomic study has confirmed its real identity, and the DNA barcoding endowed this identity with strong evidence. We conducted serial experiments of rearing the insect, calculated threshold temperatures and thermal constants, constructed reciprocal models, and made predictions. Based on coefficients such as fecundity, survival rate, sex ratio, and prediction, we estimated total numbers of females and adults reproduced by a parent female and her feminine offspring in a year, and corresponding economic losses.

## 1. Introduction

A few years before 2009, several new underground pests were found to attack American ginseng *Panax quinquefolium* L. in Liuba County, Shaanxi Province, China. Infestations of the pests caused severe damage and economic losses to local productions of the perennial, medicinal herb crop. One of the key pests was the sciarid fly, *Bradysia impatiens* (Johannsen, 1912) (Diptera: Sciaridae).

As an indispensable component of the whole project, a study of the sciarid fly was also initiated in 2009, with some observations and experiments performed in time, but suspended for a long period as the scientific name of the species was unclear till December 2021, when a supplementary experiment through the DNA barcoding of mtCOI suggested that the sciarid fly relates closely to two species, one of which was *B. impatiens*.

The first scientific name of the sciarid fly was *Sciara impatiens*, given by Johannsen in 1912, largely based on his concise descriptions of the insect and drawings of the male hypopygium and forewing (Figures 137 and 252 in his article, the same below) [1]. Roberts and Lavigne (1959) recorded an injurious effect of the fungus gnat *Bradysia impatiens* (Joh.) larvae on the growth of Kentucky bluegrass *Poa pratensis* (Linn.) and Creeping red fescue *Festuca rubra* (Linn.) sod [2]. Steffan (1966) summarized characteristics of the genus *Bradysia* Winnertz, 1867 in 380 words, and remarked “This genus can be distinguished from other North American genera by the following combination of characters: maxillary palpi three-segmented, usually with sensory pit; protibiae with preapical, ventral, unilateral comb separated from general tibial vestiture by triangular bare area, metatibiae with two subequal spurs, and posterior wing veins bare”[3]; this led to a formal transfer of *Sciara* to *Bradysia* [3,4]. As a result, the sciarid fly was moved accordingly and formed a new combination “*Bradysia impatiens* (Johannsen, 1912)” [3]. During the period from 1948 to 1978, there were still several authors who gave different names to the species, such as *Bradysia* (*Chaetosciara*) *tristicula* var. *difformis* Frey, 1948 [5] (often written as *B. difformis* Frey, 1948 later), *Sciara* (*Lycoriella*) *hardyi* Shaw, 1952, *Bradysia paupera* Tuomikoski, 1960, and *Bradysia agrestis* Sasakawa, 1978, which were all shown to be synonyms of *B. impatiens* [4]. Mohrig et al. (2013) compared the type of *Sciara impatiens* and the other specimens from the USA with the European specimens of *Bradysia difformis* Frey, and confirmed their conspecificity [4]. Johannsen’s Figure 137 [1] and Menzel and Mohrig’s Figure 95 [6] show the typical apical tooth and the isolated patch of subapical spines in identical arrangement [4].

Studies of *B. impatiens*, including previous *B. difformis* and other synonyms, have been reviewed broadly around the world [3,4,5,6,7,8,9,10,11], but few have undertaken research works in China. Here we are going to give a brief review of the hosts and infestations of the sciarid fly inside China, and look back on general approaches to identifying the sciarid fly, as well as on the impacts of different hosts and temperatures on its development and fecundity.

For the first time in 2008, Zhang et al. reported *B. difformis* as a novel and dominant species injuring various edible mushrooms in nine counties of Yunnan Province, China [12] (Table 1); they also described its biological characteristics, including four drawings such as the palpus of females, the apex of the fore tibia of males, tergite IX of males, and the genitalia of males (as imitations of [7]). Having identified 11 species in six genera based on morphotaxonomic characteristics, Shen et al. (2009) provided a key to the species in the family Sciaridae, with seven drawings from [7], and then emphasized that *B. difformis* was one of the dominant pests on edible fungi in Yunnan [13] (Table 1). Afterwards, professionals performed surveys and found that the sciarid pest injured edible and medicinal fungi, vegetable crops, ornament plants, herbages, etc., in many parts of the country [14,15,16,17,18,19,20,21,22,23,24,25,26,27,28,29,30,31,32,33,34,35,36,37,38,39] (Table 1). After identifying the sciarid fly through morphological characteristics, Qu et al. (2012) applied the mtCOI DNA barcoding technology, with analytical procedures described and the DNA sequences shown [15]. Other authors have added DNA molecular identification into their research processes if necessary (Table 1). For the first time in 2016, Xi and Hao each cited the scientific name *Bradysia impatiens* (Johannsen) of the species in their Masters theses [26,27]. Wu et al. (2016) summed up that *B. difformis* was the most common species in Gansu and Liaoning Provinces, and that its larvae bored into roots of host plants and caused severe damage to vegetables such as chive, onion, garlic, cabbage, broad bean, etc. [40]. Ye et al. (2017) reflected that former published records of *Bradysia difformis* Frey from China accorded with *Bradysia impatiens* (Johannsen), as also shown by the COI neighbor-joining tree in their paper [41]. Yang (2023) described that *B. impatiens* was an important pest on *P. quinquefolium* in Liuba, Shaanxi [37], and thus reinforced earlier observations of *Bradysia* sp. injuring the perennial medicinal herb in local areas. In their review, Gou et al. (2021) summarized the pest’s living behaviors, adult fecundity, damage caused by larvae, population dynamics, relationship between occurrence and environmental condition, and some control strategies, and pointed out existing problems to solve in the near future [42]. Although the scientific name *B. difformis* has been turned to a synonym of *B. impatiens* [4], the Chinese common name of the species, 异迟眼蕈蚊, is kept unchanged and still in use today.

Being a new insect pest on American ginseng, the sciarid fly occurs in, infests, and can even lead to outbreaks in medicinal herb plots in Liuba, Shaanxi. Facing these grim situations, we surveyed and sampled the sciarid pest, and conducted research work. Our objectives were as follows: (1) to determine its identity via morphotaxonomic study and DNA barcoding; (2) to measure and calculate its developmental threshold temperatures and thermal constants by following the method of “Thermal Sums” [43] or the “Law of effective accumulated temperature”[44], and then construct reciprocal models for developmental durations in different life stages; and (3) to test its fecundity, and moreover estimate its reproductive potential when some other coefficients are obtained. With these research tasks done properly, and with local temperature conditions addressed, we are able to increase knowledge on the biology of the species, predict its annual population dynamics, and estimate economic losses the pest would cause, so as to help ginseng farmers apply integrated pest management techniques and solve the pest problems efficiently, and to recover and promote American ginseng productivity. This article is going to present relevant processes and the results of the experimental studies.

## 2. Materials and Methods

### 2.1. Preparation of the Insect to Be Tested

Residual roots of American ginseng injured by insect pests and diseases were collected from Liuba County, Shaanxi Province, China. Living specimens of the sciarid larvae were picked out from damaged residual roots, placed within a plastic Petri dish containing wet fine sandy soils and American ginseng root powders (wet sandy dish), isolated and reared continuously at 21 °C to prepare an indoor stock colony, and then used in the following observations and experiments.

As indicated by the article’s title, the studies consist of three parts: (1) identifying the sciarid fly; (2) measuring its physiological time; and (3) testing its fecundity (the most important coefficient relating to reproductive potential; the rest of the coefficients will be given in the Results and Discussion directly).

### 2.2. Part I: Identifying the Sciarid Fly

#### 2.2.1. Observation of Body Form and Structure

More than 24 active adults, both males and females, were picked out from the indoor stock colony, fixed in Bouin’s liquid for 1 d, washed in a 75% alcohol solution, stained in 10% (*v*/*v*) dilution of Ehrlich’s hematoxylin liquid for 1 d, counterstained in 0.2% (*w*/*v*) eosin with an 85% (*v*/*v*) ethanol solution for 1 h (HE dyeing way), dehydrated in anhydrous ethanol, cleared in xylene and then mounted whole in Canadian balsam on glass slides.

Both morphological and taxonomic characteristics of the mounted adults were observed under an optical microscope equipped with a digital microphotography system (DFC 295, Leica Microsystems, Danaher Corporation, Wetzlar, Germany); microphotographs of their external forms and internal structures were taken, with sizes measured and calculated on the basis of preset scale bars.

During the same time period, living adults, eggs, larvae and pupae were observed alive microscopically, some of which were placed under a stereomicroscope (Eclipse E600, Nikon Corporation, Tokyo, Japan), and microscopic images were taken with a digital camera (Digital Sight DS-Fi1, Nikon Corporation, Tokyo, Japan). Abdomen segments are marked with Roman numerals.

#### 2.2.2. DNA Barcoding

A few larvae, pupae and adults were picked out from the indoor stock colony to perform a preliminary test. After that, each of them was used to conduct a formal experiment respectively, as follows:

(1) Chopping of cell membranes. We added an insect (a larva, a pupa or an adult of the species) into a 2 mL centrifuge tube containing quartz sands and 800 μL of CTAB solution [45,46], let the sample be shaken on a high-frequency oscillator for 1.5 min, and centrifuged it at 10,000 rpm for 15 min;
(2) Deproteinization. We placed 400 μL of the supernatant into a second centrifuge tube, added the same volume of chloroform and isoamyl alcohol mixtures (*v*/*v* = 24:1) into the second tube, and let it be centrifuged at 10,000 rpm for 15 min;
(3) Removal of polysaccharides. We placed 200 μL of the supernatant into a third centrifuge tube, added a two-fold volume of anhydrous ethanol, mixed the sample, and stored the third tube in a refrigerator at −20 °C for 12 h. We then let the sample in the third tube be centrifuged at 10,000 rpm for 10 min, and discarded the supernatant but retained the precipitate;
(4) Preparation of the DNA template. We added 200 μL of 75% ethanol to rinse the precipitate, let it be centrifuged at 10,000 rpm again for 15 min, and discarded the supernatant but retained the purer precipitate. We then let it air-dry, added in 20 μL of ddH_2_O, and then derived the DNA template;
(5) PCR amplification system, 25 μL. We took 2 μL of the DNA template, 1 μL for each of the two primers [LCO1409 (5′-GGT CAA CAA ATC ATA AAG ATA TTG G-3′) and HCO2198 (5′-TAA ACT TCA GGG TGA CCA AAA AAT CA-3′) [47], Sangon Biotech (Shanghai) Co., Ltd., Shanghai, China], 12.5 μL of Mix [The two×Taq PCR Master Mix (with common Taq polymerase), six×loading buffer, and ddH_2_O, DIYIBio, Shanghai, China], and 8.5 μL of ddH_2_O, and mixed them in a 0.2 mL PCR tube;
(6) PCR amplification. In a PCR amplifier (Type TA-40, Shanghai Hengjiu Medical Equipment Co., Britain, Shanghai, China), we let the 25 μL sample be pre-denatured at 95 °C for 5 min, denatured at 94 °C for 30 s, annealed at 55 °C for 30 s, and extended at 72 °C for 90 s, letting the process circulate 33 times;
(7) Sequencing. The PCR-amplified products (including that of larva, pupa and adult) were sent to Sangon Bioengineering (Shanghai) Co., Ltd., Shanghai, China, to determine their DNA sequences or base pairs. Sequence alignment was performed in the NCBI database, https://www.ncbi.nlm.nih.gov/ (accessed on 13 December 2021), to acquire the taxonomic status of the specimens.

### 2.3. Part II: Measuring Physiological Time of the Insect

#### 2.3.1. Experimental Design and Execution

Referring to the results of a preliminary test, and applying a completely randomized design [48,49,50], we conducted the formal rearing experiment in two batches. Six temperatures (mean ± standard deviation, or *m* ± *s*)—11.6 ± 0.4, 15.6 ± 0.1, 18.5 ± 0.1, 21.2 ± 0.1, 24.5 ± 0.1, and 27.2 ± 0.1 °C (values measured actually, the same below; refer to the fourth paragraph below for detail)—were set as treatments in Batch I of the experiment, with a total of 56 eggs reared at each of them, that is, six treatments with each having 56 replications in Batch I. Two weeks later we conducted Batch II of the same experiment—in fact a supplement and validation of Batch I. Besides repeating the six treatments stated above, we added three more temperatures of 10.0 ± 0.5, 30.0 ± 0.2, and 31.5 ± 0.4 °C as new treatments, with a total of 56, 40 or 40 eggs reared at each of the new temperatures and a total of 30 eggs reared at each of the other six temperatures, that is, nine treatments in Batch II, with each having 56, 40, or 30 replications. The concrete executive procedures are as follows:

(1) Preparation of specific “wet sandy soil paper dishes” to rear the insects in advance. Sufficient polyethylene plastic Petri dishes (9 cm in diameter) were taken as the rearing containers. Both 20 g of sterilized fine sandy soils (passed through a 60-mesh screen; aperture = 0.30 mm) and 8 mL of distilled water were added into each of the dishes and vibrated to flatten the wet sandy surface. A sheet of filter paper (about 8.2 cm in diameter) was spread on the surface. Extra water was sucked out. Seven black plastic rings (2.4 cm in outer diameter) were placed on the filter paper, and 5 mg of American ginseng root powders (passed through a 50-mesh screen; aperture = 0.36 mm) was dusted into each of the rings on the wet filter paper. These wet sandy soil paper dishes were now ready to use in the rearing experiments;
(2) Preparation of enough ≤12 h-old eggs in advance. A number of active females and males (in a sex ratio of 1:1) were sucked from the indoor stock colony with an insect aspirator and transferred into an apparatus to allow females to lay eggs inside for 12 h at room temperature (about 23–24 °C in May). The apparatus consisted of a glass beaker with a hole at the bottom that was covered by a layer of fine mesh and a wet sandy dish as the base. The former was inverted on the latter and detached, all 11 cm in diameter, and some American ginseng root powder was dusted onto the surface of the wet sandy dish. The powders were supposed to attract females to lay eggs on them, though adults were not seen to feed on these food particles;
(3) Inoculation, rearing, and observation. In Batch I of the experiment, a total of 24 wet sandy soil paper dishes were used, and two of the ≤12 h-old eggs were inoculated into each of the rings with a piece of hair under a stereomicroscope. The 24 wet sandy soil paper dishes with eggs inoculated were divided into six groups randomly. Containing four wet sandy soil paper dishes or 56 eggs, each group was then placed into a thermostatic cabinet (Type 250D, Jintan Jerier Electrical Appliance Co., Ltd., Changzhou, China) at a definite temperature to rear for a generation (from egg to adult). The conditions in Batch I, were identical in Batch II except that only one ≤12 h-old egg was inoculated into each of the rings, with a single individual rearing implemented. The developing statuses of eggs, subsequent larvae and pupae in each plastic ring, each wet sandy soil paper dish, and each cabinet were observed and recorded under a stereomicroscope twice every day at 7:30–8:30 and 19:30–20:30 till the emergence of all the filial adults.

To ensure the factuality of the treatments, we measured each of the temperatures at the exact points where we were rearing the insects from egg to adult in each of the thermostatic cabinets with a temperature and humidity meter (Type TES-1361C, TES Electronic Industry Co., Ltd., Taiwan, China; calibrated with a national second-class mercury thermometer before use) in an imitational way. The digital number displayed on the temperature controller built into each thermostatic cabinet was only regarded as a reference of temperature to be used to check if the cabinet was running stably, and was not used for the following statistical analyses. The actual temperatures measured are given above; the RH was 95–100% within the space above the wet filter paper inside all the wet sandy soil paper dishes. All the cabinets were placed in shaded rooms, with very weak sunlight diffused through the front glass window, and shifted naturally with day and night.

#### 2.3.2. Collation and Statistical Analyses of the Raw Data

The raw data derived from the six treatments at 11.6, 15.6, 18.5, 21.2, 24.5 and 27.2 °C in Batch I of the experiment were combined with those in Batch II based on the same temperature, with their sample sizes enlarged and relative stabilities increased. The raw data from the two treatments at 10.0 and 30.0 °C in Batch II were retained but the one at 31.5 °C was discarded due to all the inoculated eggs having died of heat. Thus, the original 15 treatments in Batches I and II were then combined into eight valid ones.

Based on the raw records, the raw data regarding insect development in egg, larval and pupal stages were accepted only if the insect had completed a generational development and emerged as a new filial adult; corresponding raw data were disused if the inoculated egg or the subsequent larva or pupa died before maturing. In each of the eight valid treatments, the accepted raw data themselves formed a dataset for the whole immature stage, which was then divided into three subsets according to the insect’s egg, larval and pupal stages. The three subsets and the dataset denote the actual developmental durations in days of the insect in its egg, larval, pupal and immature (egg + larva + pupa) stages; the mean and standard error (*m* ± *SE*) of each subset and the dataset were calculated, respectively [48,50].

##### Analysis of Variance of the Actual Developmental Durations

The survival rate of the immature individual in each of the eight valid treatments was calculated (= no. new adults/no. inoculated eggs × 100%). Grouped into each of the life stages of the insect, the eight valid treatments and the raw data in corresponding subsets or datasets were subject to an analysis of variance (ANOVA, for a randomized design with different sample sizes) [48,50]. Given that the obtained *F*-test ratios showed significant differences among treatments (*p* < 0.01), multiple comparisons between the means of different treatments were performed, and the means and their significance were output to express the results [48,50].

##### Correlation and Regression Analysis Between Developmental Durations and Rearing Temperatures

According to the “Thermal Sums” equation, *N*(*T* − *C*) = *K* [43], after obtaining the means of the actual developmental durations (*N*) and rearing temperatures (*T*) of the insect in its four life stages, we calculated its developmental threshold temperature (*C*) and thermal constant (*K*) in each stage by solving the equation with a linearized regression analysis.

The first seven of the eight valid rearing temperatures (10.0, 11.6, 15.6, 18.5, 21.2, 24.5 and 27.2 °C; excluding 30.0 °C, see explanations in the Section 3 and Section 4 for detail) were taken as one of the two variables (*T*), and corresponding means for actual developmental durations of the insect in its four life stages as the other (*N*). It is known that the statistical relationship between *N* and *T* is nonlinear, thus a linearizable nonlinear regression analysis was applied. To meet the requirements of linearization, variable *N* was transformed to its reciprocal, thus becoming growth rate or developmental velocity (*V*, = 1/*N*). Under the linearized condition, we fit a linear correlation and regression analysis to variables *T* and *V*, calculated a linear correlation coefficient (*r*) and built a simple regression equation *T* = *a* + *bV* in each life stage. The intercept *a* and the slope *b* in the linear equation (in terms of regression analysis) are equal to *C* and *K* (in terms of biology or physiology, the same below); therefore, the linear equation *T* = *a* + *bV* is an equivalent of *T* = *C* + *KV*. The standard errors of the intercept and the slope were calculated accordingly, with *s_C_* = *s_a_* and *s_K_* = *s_b_* [48,50].

Furthermore, when *V* was transformed back to *N* and the simple equation was rearranged, a real reciprocal model was constructed, *N* = *K*/(*T* − *C*), whose track is curvilinear. The theoretical means of *N* in each life stage were estimated by use of a corresponding reciprocal model, and a chi-square goodness-of-fit test was performed between the means observed and estimated to derive the fit probability of the reciprocal model [49]; the trends of the means observed and estimated were plotted against rearing temperatures to illustrate the tracks for the developmental durations of the insect in its four life stages.

### 2.4. Part III: Testing Fecundity of the Insect

In conjunction with the experiment of “Measuring Physiological Time of the Insect” and with the help of the apparatus, we tested the fecundity of females after inoculating those ≤12 h-old eggs into rings of Batches I and II, respectively. The residual eggs in each egg mass were counted in the wet sandy dish of the apparatus, and removed but no longer used. The adults sucked out previously from the apparatus were retransferred back into it, and reared till all of them died; a count of eggs laid before their death was then taken. All the eggs, used and disused, were summed together, and so were all the females used; then an average fecundity of the insect was calculated. In addition, a few extra observations were conducted randomly, and an overall average fecundity was estimated.

## 3. Results

### 3.1. Taxonomic Position of the Species

#### 3.1.1. Morphotaxonomic Characteristics

##### Adult

In Johannsen (1912), we can read traits of the genus *Sciara* Meigen, 1803 [1]. Following the key to species in the genus given by him, we find the species *Sciara impatiens* and its characteristics [1], as follows:

Table of Species: … aa. Medium or small species… bb. Media and cubitus without setae… cc. R_1_ ends at least 1/16 of the wing length proximad of the forking of M; the base of Rs is distad of the midpoint between the humeral crossvein and the tip of R_1_… dd. Black or fuscous species… ee… ff… gg… hh. Petiole of the cubitus at least half as long as the basal section of M… ii. Rs ends distad of 0.85 of wing length… jj… kk. Clasper with 5 or more apical and subapical setae or spines… l. Apex of clasper with 6 or 8 setae of which one is somewhat set apart from the others (Figure 137); halteres yellow; hind coxae dark; wing (Figure 252). 45. *impatiens* n. sp.

45. Sciara impatiens n. sp.

Male. Length 1.25 mm. Fuscous, including antennae which are less than 1/2 as long as the body, intermediate segments being but little longer than broad; palpi yellow. Thorax sub-opaque; hypopygium dark, clasper (Figure 137). Coxae and legs yellow, tarsi darker, hind tarsus slightly shorter than the tibia. Wings grayish hyaline, veins dark, strong (Figure 252). Halteres fuscous. Bred from larvae found in earth adhering to the roots of *Impatiens*. Ithaca, N. Y. [1].

In addition to the record of Frey (1948) [5] and the work of Steffan (1966) [3], Menzel et al. (2003) redescribed this sciarid fly under the name *B. difformis* in detail; in particular, they provided 10 free-hand drawings to characterize the species [7]. Our observations on specimens of the sciarid fly accord with the characteristics reported above in most cases. We are going to provide some more supplementary expressions and quite a few microphotographs to confirm the identity of the species.

Body form. Images of the adult body are shown in Figure 1 (male) and Figure 2 (female). The length of males is (*m* ± *s*) 1.93 ± 0.14 mm (*n* = 7; ext. 1.63–2.02 mm); that of females is 2.05 ± 0.23 mm (*n* = 9; ext. 1.83–2.50). Color distortion occured on the slide specimen (Figure 1), changing to brown and/or yellow from fuscous or black (Figure 2), meaning a description on colors is effective only if the specimen is observed alive; otherwise, it is no longer true, especially on a glass slide specimen, and the same is true below.

Head**.** Ovoid, as wide as mesonotum, black and/or fuscous (Figure 1, Figure 2 and Figure 3); a pair of compound eyes, connected with an eye bridge, three facets wide (Figure 3 and Figure 4); three ocelli on prefrons; a pair of antennae, with each consisting of a scape, a pedicel and 14 flagellomeres; the fourth flagellomere about 1.5 times as long as it is wide (Figure 4); a pair of maxillary palpi, with each three segmented (Figure 5).

Thorax. Moderately arched, compact, and wrapped by quite a few sclerites with definite names (Figure 3 and Figure 6); antepronotum and postpronotum small; mesonotum big, covered with sparse bristles, nearly square when viewed dorsally; followed by a half-moon-shaped scutellum with three or more stronger bristles pointing diagonally upwards; lateral sides of mesothorax and metathorax protected by katepisternum, laterotergite, mediotergite, metanotum, etc. (Figure 6).

Fore tibia with a spur and a comb-like row at distal end (Figure 7A,B); mid and hind tibiae without any comb-like row but with two subequal stout spurs (Figure 7C). Each tarsus with five segments and a pair of tarsal claws, not toothed (Figure 7D).

The length of the male forewing is (*m* ± *s*) 1.67 ± 0.49 mm (*n* = 4; ext. 0.93–1.98); that of the female is 1.78 ± 0.34 mm (*n* = 9; ext. 1.42–2.26). Costa and radius veins are strong, with two to three rows of macrotrichia along the costa, and one row along the radius; other wing veins such as the media with a fork (including M_1_ and M_2_), the cubitus with two forks (including CuA_1_, CuA_2_ and CuP), the analis (2A) and the radiomedial cross vein (r-m) are normal (Figure 8A,C,D); the wing surface is covered with dense microtrichia (Figure 8B); the stem of the media vein is weak (Figure 8C). The venation is quite similar to that in Figure 252 [1].

Abdomen. Consisting of nine segments; segments II–V are bigger than the rest; segment IX and its appendages are specialized into a hypopygium in male (Figure 9 and Figure 10) and an ovipositor in female (Figure 11). Tergite IX (epiproct) of the male is connected with a pair of gonocoxites and gonostyles (claspers), which enclose the anus, a pair of cushion-like cerci with setae, and a tegmen holding the aedeagus (Figure 10). The apex of gonostyle with thicker bristles, among which is an apical tooth; about five to seven subequal curved spines directed ventromedially. The epiproct of the female is invisible in Figure 11A,B, but the periproct is apparent, and it originates from segment VIII and covers the basal part of cerci. The periproct is followed by a pair of cerci afterwards, each with two segments that are visible (Figure 11B,C).

Based on our observations and the microphotographs taken, and following comparisons with the texts and drawings given by Johannsen (1912) [1], Menzel et al. (2003) [7] and Mohrig et al. (2013) [4], we can identify the sciarid species collected from Liuba, Shaanxi, as *Bradysia impatiens* (Johannsen, 1912), because *Sciara impatiens* and *Bradysia difformis* are all synonyms for the real identity [4].

##### Egg, Larva and Pupa

*Bradysia impatiens* is holometabolous. Its egg is ellipsoid and semitransparent when just laid (Figure 2 and Figure 12), with a size of (*m* ± *s*) 0.236 ± 0.009 × 0.148 ± 0.007 mm (*n* = 211). Females deposit often in strings (Figure 2) and/or egg masses (Figure 12), and sometimes sporadically.

Larva, apoda, eucephalous, 1.5–5.0 mm long (matured), with a black head and chewing mouthparts; there are 12 segments visible in its carcass (trunk) (Figure 13). Larvae can spin silk, weave thin nets, and feed and live under the nets; as stated above, they are both saprophytic and parasitic, with a lot of host plants and edible and medicinal fungi. A matured larva makes a simple pupal chamber near the feeding site and pupates inside.

Pupa, obtect, crescent-shaped, with a size of 1.8 mm long and 0.6 mm wide, and with a black cephalothorax and grayish-white abdomen that has nine visible segments; each of segments II–VII has a spiracle (Figure 14).

The injury of American ginseng caused by *B. impatiens* takes place when the insect is in its larval stage. Female adults lay eggs inside and/or outside the roots of the host plant in the field. After hatching from their eggs, the larvae bore into the tender roots of seedlings and feed on the tissues inside (Figure 15A), consuming nutrients and causing severe damage to seedlings, even sometimes ruining the whole plot of American ginseng (Figure 15B). Compared with vigorous seedlings in protected plots (Figure 15C), seedlings that are injured by the sciarid pest are bound to wilt and die (Figure 15B), losing economic value in great measure.

#### 3.1.2. DNA Barcoding

The three DNA sequences (Figure 16, in part) that were sent back from Sangon Biotech (Shanghai) Co., Ltd. were compared with those in the database of the National Center for Biotechnology Information (NCBI), https://www.ncbi.nlm.nih.gov/, (accessed on 13 December 2021); they were matched to both species *Bradysia impatiens* and *B. ocellaris*. Compared with the DNA sequences of the larvae, pupae and adults of *B. impatiens* given by the accession MT357556.1, the accession length of the DNA strands was 658 base pairs, and the percent identities of the tested specimens were larvae 96.72%, pupae 99.18%, and adults 96.71%, whereas compared with those of *B. ocellaris* given by KU379822.1, possessing a similar accession length, the percent identities became larvae 95.89%, pupae 100.00%, and adults 96.22%. There were some more results showing similar trends, indicating that the percent identities were only slightly different from each other regardless of whether the tested larvae, pupae and adults were *B. impatiens* or *B. ocellaris*.

The results of DNA barcoding in the mtCOI gene provide strong evidence of it being *B. impatiens*, though as shown by the comparison, the species *B. ocellaris* could have DNA sequences that are very similar to those of *B. impatiens*. Based on both DNA barcoding and morphotaxonomic identifications, we can recognize again that the species being tested is *Bradysia impatiens* (Johannsen).

All the following descriptions produced by our team are given under the scientific name, *B. impatiens*.

### 3.2. Physiological Time

#### 3.2.1. Developmental Durations of *B. impatiens* in Different Life Stages

The results of ANOVA show that there were significant differences (*p* < 0.01) among the means for the developmental durations of *B. impatiens* in its egg, larval, pupal and immature stages within the temperature ranges of 10.0 ± 0.5 to 30.0 ± 0.2 °C (Table 2). All the eggs that were inoculated in the treatment at 31.5 ± 0.4 °C died, indicating that this temperature exerted a completely lethal heat effect to the insects tested, and leading to an outcome whereby there were no data to compare with those of the other eight valid treatments.

In the temperature range from 10.0 ± 0.5 to 21.2 ± 0.1 °C, the differences between any pair of average durations within the columns of egg, larva, pupa and immature were larger, and all of them were significant at *p* < 0.01, except that there was a small change in significance in the column of larva (Table 2). In the range from 21.2 ± 0.1 to 30.0 ± 0.2 °C, the differences between any pair of average durations became relatively smaller, with the differences significant (*p* < 0.05) when the average durations were compared with each other except in the column of egg (Table 2).

Attention should be paid to the fact that the average duration of larva at 30 ± 0.2 °C (12.2 ± 0.26 days) was greater than that at 27.2 ± 0.1 °C (11.3 ± 0.54 days), which led to a similar trend whereby the average duration of immature insects at 30 ± 0.2 °C (18.3 ± 0.32 days) was slightly greater than that at 27.2 ± 0.1 °C (18.2 ± 0.53 days) (Table 2). This phenomenon revealed that, at 30.0 ± 0.2 °C, the temperature did not promote but retarded the development of the insect tested, though the difference was small.

Generally speaking, the experiment has shown that the developmental durations of the insect in its four life stages decreased rapidly as the rearing temperatures rose, suggesting that its developmental rate *V* increased in the meantime, but with an exception at 30.0 ± 0.2 °C. This fact does not seem strange, since a higher temperature caused the eggs to die, as shown in the treatment at 31.5 ± 0.4 °C. It is apparent that the data point at 30.0 ± 0.2 °C is a departed outlier, and was thus discarded. As a result, the other seven data points derived at 10.0 ± 0.5 to 27.2 ± 0.1 °C in each stage were retained to calculate the threshold temperature and the thermal constant by fitting a linear correlation and regression analysis.

As seen in Table 2, the *SE* for the average durations of eggs and pupae were relatively smaller, indicating that their durations were more stable. On the other hand, the *SE* for those of larvae was larger, suggesting that the *SE* for those of immature insects expanded too. The larger *SE* would indicate that the 95% confidence intervals for average durations of larvae and immature insects were wider than those of eggs and pupae. Furthermore, the survival rates of these immatures insects from egg to new filial adult ranged from 14.3% to 58.1%, with a mean of 46.4% (= 284/612; Table 2); the lowest rate was found at 10.0 ± 0.5 °C (14.3%), and the rates in the rest of the treatments ranged from 40.7% to 58.1%, showing that the temperatures tested, especially the lowest and the highest ones (10.0 and 31.5 °C), had great impacts on the survival of the insects. The raw records show that many first-instar larvae disappeared or died of starvation in all the treatments except at 31.5 °C; however, their mortality dropped quickly when living first-instar larvae molted and grew into second-instar larvae and beyond.

#### 3.2.2. Threshold Temperatures, Thermal Constants, and Reciprocal Models

After the data point at 30.0 ± 0.2 °C was discarded, the linear correlation analysis of the remaining seven data points in each stage indicated that *T* was closely related to *V* (*p* < 0.001) (Table 3). Calculated from a linear regression analysis between *T* and *V*, the developmental threshold temperatures (*a* or *C*) of the insects in their four life stages ranged from 5.5 to 6.8 °C (Table 3), indicating that the insect, especially in the egg stage, could sufficiently utilize environmental heat to grow and develop; the thermal constants (*b* or *K*) ranged from 69.9 to 374.9 °C (Table 3), indicating that the eggs and pupae were able to rely on relatively less heat to complete growth and development in relatively shorter durations, but the larvae required more heat to do so, because they had to feed for a longer time, accumulate enough nutrients, and prepare for pupation and eclosion as well as reproduction in the adult stage (Table 3).

The reciprocal model, *N* = *K*/(*T* − *C*), is derived when *V* is transformed back to *N* and the linear regression equation, *T* = *a* + *bV* or *T* = *C* + *KV*, is rearranged. The chi-square goodness-of-fit test has indicated that each of the *χ*^2^ values was very low, meaning the probability that the estimated average durations *NE* fit the observed points *NO* was larger than 0.9996 (Table 3); therefore, we have concluded with the null hypothesis *H*_0_, that the reciprocal models fit the developmental duration trends of eggs, larvae, pupae, and immature insects (Table 3). When the standard errors of *K* and *C* are added into the equations, the formula becomes *N* = (*K* ± *s_K_*)/[*T* − (*C* ± *s_C_*)], and the four real reciprocal models become

*N_egg_* = (69.9 ± 4.37)/[*T* − (5.5 ± 0.90)]; (1)

*N_larva_* = (230.1 ± 5.39)/[*T* − (6.8 ± 0.31)]; (2)

*N_pupa_* = (75.1 ± 2.54)/[*T* − (6.7 ± 0.45)]; and (3)

*N_generation_* = (374.9 ± 8.15)/[*T* − (6.6 ± 0.29)]. (4)

Figure 17 reflects the curvilinear descending tracks for the developmental durations of the insect in its four life stages, which rapidly shorten with rearing temperature increases.

### 3.3. Fecundity of B. impatiens

Adults mate soon after eclosion, and females lay eggs often in strings or in egg masse, as shown in Figure 2 and Figure 12. There were 2 to 42 eggs in the egg masses counted, with a mean of 16.8 eggs (*n* = 49). We tested using apparatus and examined randomly; the overall average fecundity was 59.2 eggs per female under favorable laboratory conditions. Based on an actual observation, the sex ratio of female to male was about 2:1 (*n* = 85), with the female portion equaling 2/3 or 0.667.

## 4. Discussions

### 4.1. Identity of the Insect Specimens—B. impatiens

Referring to the “Key to Genus in Sciaridae” proposed by Yang et al. (1993), and going along the entries, “Only wing veins C, R, R_1_, and Rs with macrotrichia”, “Distal end of fore tibia with a comb-like row”, “Number of spurs on distal tibia of three pairs of legs being 1–2–2”, and “Cross vein r-m and stem of M without macrotrichia”[51], we meet with the genus *Bradysia* Winnertz, 1867, to which the sciarid fly being studied belongs. Genus *Bradysia* contains medium or small species that were previously placed in the genus *Sciara* Meigen, 1803, a phylogenetically ancient genus of the family Sciaridae, and includes the earliest described sciarid species, which is obviously related to their remarkable appearance, especially their large size (up to 6.7 mm in length for adult males and 8.1 mm for females) [52]. The hypopygia of males in *Sciara* [52] look entirely different from those in *Bradysia* [7,51]. Another prominent trait is that the media and cubitus veins have macrotrichia in *Sciara*, but not in *Bradysia* [51].

After comparing our microphotographs on the sciarid fly with the free-hand drawings and redescriptions given by Menzel et al. (2003) [7], we wondered if the specimens were really *B. difformis,* because the comparison disagreed with the results of DNA barcoding. The doubt was not solved until we found and read the article published by Mohrig et al. (2013), who determined that *Bradysia impatiens* (Johannsen) is a real species, whereas *B. difformis* is only one of its synonyms [4].

Every species has a specific trait or diagnostic characteristic that distinguishes it from others. The male hypopygium, with the distinct arrangement of an apical tooth and five to seven subapical spines, is such a trait that *B. impatiens* possesses at the species level. Other traits, such as the ratio of length to width of the fourth flagellomere, the sensory pit and setae on the maxillary palpus, the comb-like row on the distal end of the fore tibia, the venation and macrotrichia arrangement, the shape of tergite IX and the setal arrangement, are supplementary ones that assist one in fulfilling morphological taxonomic studies. In addition, we have provided many microphotographs and permanent records, which would provide professionals with more biological characteristics of *B. impatiens* in terms of both exterior forms and interior structures. The experiment on DNA barcoding demonstrated at a molecular level the results of our morphotaxonomic study, as both were complementary.

There is still a question to ask and answer, namely, what is the actual body length of a living adult male? It was stated to be 1.25 mm in Johannsen (1912) [1], but 1.8–2.1 mm in Menzel et al. (2003) [7], and it was 1.93 ± 0.14 mm (*m* ± *s*; *n* = 7; ext. 1.63–2.02 mm) in our own measurement, which is very close to that of Menzel et al. (2003) [7]. After having reviewed the species *Bradysia impatiens*, Mohrig et al. (2013) wrote an explanatory note down: “[2 slides; hypopygium not in good position, wing; body, antennae missing]; hereby designated in order to fix the name” [4]. The phrases “body, antennae missing” meant the target specimen(s) were incomplete. It is known that body length is not a decisive but a referential trait. The length 1.25 mm was much smaller than those of specimens of the same species that we have seen, which reminded us to read the original article carefully. In Johannsen (1912), we did find such a sentence as “…the body length given refers to dried specimens, balsam mounts and alcoholic specimens being about a third longer” [1]. The product of 1.25 mm × (1 + 1/3) is 1.67 mm, which is slightly longer than the shortest length, 1.63 mm, in our measurement listed above, pointing to a wild specimen collected from a damaged residual root of American ginseng. Thus the corrected body length, 1.67 mm, for a living male is believable, and the extent 1.8–2.1 mm [7], or 1.93 ± 0.14 mm, for the body length of living adult males is representative.

### 4.2. Physiological Time of B. impatiens

Threshold temperature and thermal constant are two coefficients used in the regression analysis of plants and poikilotherms growing and developing; in other words, they are a measure of physiological time [53]. Their calculations are based on the equation for “Thermal Sums”, *N*(*T* − *C*) = *K*, contributed by Réaumur and Gasparin [43]. To predict plant development, Réaumur (1735) introduced the concept of the thermal constant and formulated it as *NT* = *K*, where *N* represents the time required for a growth period, *T* denotes the average temperature during this period, and *K* is the thermal constant [54]. Since the growth rate *V* = 1/*N*, the formula may be rewritten as *V* = *T*/*K* [43]. Gasparin (1844) proposed the concept of effective temperature, leading to the need to determine the developmental threshold temperature (*C*); thus, the formula *NT* = *K* is revised as *N*(*T − C*) = *K* [55], which is now a commonly used formula for representing the total effective temperature required for insect development within a certain temperature range [43]. Simpson (1903) developed this formula further by taking “day-degree” as the unit of the thermal constant [43,56]. Depending upon the same theoretical basis, scientific workers have proposed several methods, two of which are at least commonly employed to calculate coefficients *C* and *K*.

Method 1: As applied in the Section 2, after obtaining *N* and *T* from serial experiments of rearing insects, we began by calculating variable *V* (the growth rate, or the reciprocal of *N*), and fitting the linear regression equation *T* = *C* + *KV* to the first seven data points in each life stage (Refer to the section for detail).

Method 2: This contains the following procedures. (1) Start with the formula *K* = (Days)(*T* − *Tth*), where *K* is a thermal constant, *T* is air temperature in °C and *Tth* is a threshold temperature (= *C* in Method 1). (2) Designate the rate of growth (*R*, = *V* in Method 1) as the dependent variable and air temperature (*T*) as the independent variable. (3) Fitting linear regression to the data points, excluding all points that depart strongly from the line at high temperature, *R* = *a* + *bT*, where *a* and *b* are the intercept and slope, respectively. (4) Calculate *Tth* and *K*; *Tth* = −*a*/*b*, and *K* = 1/*b* [53].

To verify the effect of Method 2, we followed its steps one by one, namely, fitting the linear regression equation *R* = *a* + *bT* to the same seven data points in each life stage, and calculating *Tth* via −*a*/*b* and *K* via 1/*b*. In Table 4, the results indicate the following: (1) All the linear correlation coefficients (*r*) are significant at *p* < 0.001, and exactly the same as those in Table 3. (2) Coefficients *Tth* and *K* are very close to those in Table 3, except that the *Tth* of eggs is 0.2 °C lower than the *C* shown in Table 3, and the *K* of eggs is 1.4 d°C higher than that in Table 3, because its *r* value (0.9904) is lower than the rest (0.9971–0.9988) (Table 3 and Table 4). It is not difficult to show mathematically that *Tth* and *K* in Table 4 would be exactly the same as *C* and *K* in Table 3 only if *r* = 1, that is, an absolute or complete correlation would have existed between the two variables *T* and *V*, or *R* and *T*.

The verification demonstrates that both Methods 1 and 2 have similar effects in outputting coefficients *r*, *C* or *Tth*, and *K*, though Method 1 does not allow us to tell the dependent variable from the independent one in the calculating process. Compared with Method 2, by which one calculates *Tth* and *K* in a complicated way, using Method 1 one can obtain *C* and *K* directly. Meanwhile, one can calculate their standard errors *s_C_* and *s_K_* based on the serial formulae established for linear regression analysis [50]. All the rest of the procedures, such as transforming *V* back to *N*, constructing reciprocal models, and confirming their probabilities of fit to the data points observed, are bound to become relatively easier afterwards. Therefore, using Method 1 is recommended for performing similar analyses.

Usually, Method 1 ends at the step when the calculation of coefficients *C* and *K*, plus sometimes their *SE*, has been finished. However, authors have already developed methods by constructing reciprocal models, and published some articles with examples, such as the one for the flea beetle, pupal *Phyllotreta vittata* Fabr. [57]; the one for the worm *Enchytraeus buchholzi* Vejdovský (corrected name) [58]; the one for American ginseng *Panax quinquefolium* L. in its early seedling stage [59]; and the one for the root mite *Schwiebea similis* Manson [60]. The present one used for the sciarid fly *Bradysia impatiens* (Johannsen) is just a brand new application of the theory and methodology. The name, “Reciprocal model”, is given because *V*, the reciprocal of the dependent variable *N*, participates in regression computation, and the independent variable *T* is part of the denominator in the real equation.

There are two types of thermal thresholds, namely, lower and upper temperature thresholds. Coefficient *C* or *Tth* refers to the lower temperature threshold, which can be extrapolated via a linear regression analysis but never actually bio-assayed. We have calculated and discussed *C* or *Tth* above, and we are going to discuss the upper temperature threshold below.

Elkinton (1993) summarized that “Insects are poikilothermic animals… As temperature rises, so does rate of growth, until some optimum temperature (*T_opt_*) is reached. At higher temperatures, the rate of growth declines dramatically, and above this range (*T_max_*) the insect will die. However, most species are well adapted to the environment in which they live and rarely experience temperatures above *T_opt_*” [53]. In our serial rearing experiments, the start point of the *T_opt_* was observed at 27.2 °C, which should range from 27.2 °C to a point lower than 30.0 °C, the maximum temperature (*T_max_*), at which development of the sciarid fly was retarded; consequently, applying 31.5 °C, a temperature above the *T_max_*, caused the eggs inoculated to die of heat. Zhu et al. (2018) found that *B. odoriphaga* and *B. difformis* showed slow developmental rates and low survival rates and fecundity when the temperature was higher than 30 °C [30], which accords with our observation. The facts demonstrate that the *T_max_* is the upper temperature threshold.

In the temperature peak period in Zhakoushi Village (alt. 1722 m), the air minimum, mean and maximum temperatures were 14.1, 18.7 and 25.3 °C, respectively, and the soil minimum, mean and maximum temperatures were 17.1, 17.8 and 18.9 °C, respectively [61]. These are all lower than the *T_opt_* (27.2 °C) given above, which agrees with Elkinton’s summation.

As regards the effects of temperatures on the development and fecundity of *B. difformis*, Liu et al. (2015) reared the insects with fake stems of Chinese leek *Allium tuberosum* at constant temperatures of 10, 15, 20, 25, and 30 °C, respectively, tested developmental durations of the insects in their egg, larval, pupal and egg–pupa (immature) stages, and calculated their threshold temperatures and thermal constants via Method 1 [23]. Compared with our results, as shown in Table 3, the coefficients *C* and *K* derived from their experiment—5.8 °C and 218.0 d°C in the larval stage and 5.0 °C and 395.8 d°C in the immature stage (having been rounded to one decimal according to actual accuracy)—might be acceptable biostatistically, but the rest of the values departed far from ours, perhaps because they neither conducted residual analyses nor identified and discarded possible outlier(s) [49].

As listed in Table 3, coefficient *C* values of the eggs and larvae of *B. impatiens* were 5.5 and 6.8 °C, whereas that of American ginseng was 3.7 °C in its early seedling stage [59]; the former are only 1.8 and 3.1 °C higher than the latter, meaning that eggs of the sciarid pest would be able to initiate their development under relatively lower environmental heat, and larvae would be capable of feeding on fresh tissues of the host plant in early spring no sooner than their revival from an overwintering state in the field.

Estimating with the reciprocal models, one may foresee developmental phases of eggs, larvae and/or pupae of *B. impatiens* in the field if one has sampled properly, and can thus accomplish strategies of integrated pest management. The reciprocal model for its generational development can also be applied to predict population dynamics of the sciarid pest in one year. Based on the polynomial regression models for periodical temperature curves (also called Fourier’s curves) established within four plastic greenhouses of American ginseng located in villages Zaomulan (alt. 1236 m) and Zhakoushi (alt. 1722 m), Liuba County [61], we computed local effective accumulated temperatures above 6.6 °C (EAT) in the soil at 10 cm depth, where the host plant stretches its roots on which the larvae may feed. The resulting EAT was 2137.5 d°C a year in Zaomulan and 1574.5 d°C a year in Zhakoushi. When these two values of EAT are divided by the thermal constant for generational development (*K*, = 374.9 d°C, Table 3), the quotient equals 5.7 and 4.2, meaning that *B. impatiens* is going to reappear for 5.7 generations in American ginseng fields in Zaomulan, and for 4.2 generations in Zhakoushi, each year.

There are two independent on-site investigations that prove this method and its predictions to be valid and applicable, as follow below.

Zhong et al. (2018) showed that *B. difformis* overwintered in mixed populations of larvae and pupae in soils at 10.0–24.5 cm (15.0 cm on average); the fractions of larvae from the first to the fourth instar were 2.1%, 20.1%, 58.2%, and 12.7%, and that of pupae was 6.9%; the species occurred for five generations a year in open leek fields in Dongling district of Shenyang, Liaoning [29]. The annual air mean temperature in Shenyang was 8.4 °C [62], which is slightly higher than that in Zhakoushi (8.3 °C), Liuba County [61]. This comparison of the annual air mean temperatures between the two regions implies that their environmental heat conditions were similar; thus, their observation provides our prediction with general and practical support.

*Bradysia impatiens* was observed to overwinter as mixtures of pupae and larvae within roots of American ginseng in Zhakoushi Village (alt. 1653 m), too [37]. With sticky insect traps at Site 1 in Zhakoushi, Yang (2023) captured quite a few adult *B. impatiens* from early April to mid-May; after that, she trapped more adults on May 27, June 10, July 15 and August 12, and fewer ones on September 9 and 23 (Figure 18) [37]. The trend in adults trapped at Site 2 in the same village is similar to that for those at Site 1, but their number was much lower (Figure 18).

As shown in Figure 18, the occurrence of Generation 1 (G1) adults was first recorded on April 8 in the year [37]. These G1 adults must have emerged from pupae that had previously been overwintering at Site 1 in Zhakoushi (alt. 1653 m), and crawled from American ginseng roots in soils deeper than 10 cm underground up to the surface to mate and reproduce. Meanwhile, the overwintered larvae were revived around March 27, when the soil mean temperature at 10 cm reached 6.6 °C in Zhakoushi [61], and moved from deeper regions to upper layers, say, 1–5 cm depth. In this way they entered into new microhabitats to absorb more heat, feed on fresh root tissues, and grow and develop quickly.

The population dynamics of adult *B. impatiens* were found to be related to thermal dynamics in Zhakoushi Village (Figure 18). To explain this phenomenon, we need to know about the temperatures in soil layers at a 1–5 cm depth because most larvae live in their microhabitats except when in the overwintering stage, and they cause damage to American ginseng from April to September. We can estimate temperatures in these microhabitats based on the information presented below, though they have not been directly measured before.

Three periodical temperature curves were abstracted from our meteorological studies [61] and recombined with other information to form Figure 19, which illustrates the following: (1) From March to August, the trend of the air mean temperature (AMT, estimated; the same below) was similar to that of the soil mean temperature at 10 cm depth (SMT_10 cm). (2) The air maximum temperature (AMAX) curve exhibited a trend nearly similar to that of the AMT curve, but remained about 7.2 °C higher than that of the SMT_10 cm curve, which means the solar radiation heated the land surface via the atmosphere, causing the AMT and SMT_10 cm to rise. Yang’s sample sites (alt. 1653 m) [37] were 69 m lower than ours (alt. 1722 m) [61]; this decrease in altitude could only cause the SMT_10 cm curve to rise about 0.07 °C, which is negligible.

In a whole day, the temperature at different times varies along a sine curve, and the areal integral of half its cycle equals 2. The length of the baseline of half its cycle is π, so the quotient of 2/π is 0.637, equal to the height of the rectangle transformed from half the cycle of the sine curve. Let the semi-amplitude 7.2 °C be multiplied by 0.637; the product is 4.6 °C, functioning as an equivalent to cover the ground with warm air that is 4.6 °C higher than the SMT_10 cm curve for half a day. Upper layers of the soil then get warmer in the day time, but lose some heat at night. Since the increase in the heat in the air and the soil is greater than the loss in spring and summer, both curves of the AMT and the SMT_10 cm rose continually from February to July in the field (Figure 19). Using interpolation, the temperatures at 1, 3 and 5 cm depths were 4.1, 3.2 and 2.3 °C higher than the SMT_10 cm in the daytime, respectively. When the SMT_10 cm curve was taken as a skeleton, an empirical average of 2.3 °C was added to each daily mean of the curve from March 1 to May 29, 1.9 °C from May 30 to August 31, and 1.3 °C from September 1 to 30, accordingly (Figure 19). After the additions were completed, an adjusted curve was obtained, namely, SMT_1–5 cm, which would reflect the trend in the SMT at the 1–5 cm depth (Figure 19), and was ready to use in estimating occurrence dates of adults in subsequent generations.

There are seven data points from April 8 to May 20 in Figure 18 showing the numbers of G1 adults captured. Taking their occurrence dates as starting points of the next generation, subtracting the threshold temperature (*C* = 6.6 °C; Figure 19) from the daily mean of the SMT_1–5 cm curve first, and then summing the daily difference along the curve till meeting the thermal constant (*K* = 374.9 d°C), we may estimate the dates at which G2 adults will occur, and their range is from May 27 to June 28. In the same way, we may also estimate the dates at which G3 and G4 adults appear, and the result ranges from July 3 to 28 and from August 2 to 27, respectively (Figure 19). Compared with the ranges of dates wherein the bigger peaks are located in Figure 18, our estimated ones almost coincide with them. As regards the two relatively smaller peaks in September, they should be taken to represent G5 adults, accounting for 20% or more of the populations, whereas the rest of the populations containing pupae and larvae are going to move downwards into deeper parts of the host roots to overwinter.

The analysis and comparison given above show the following: (1) Based on both the warmer air on the land surface and the curve of the SMT_10 cm, we may adjust and draw a new curve of the soil mean temperature in 1–5 cm layers underground, SMT_1–5 cm, using mathematical methods. (2) According to this new curve and the species’ physiological time, we may estimate occurrence dates of adult *B. impatiens* in the field. The estimated occurrence dates are basically consistent with the actually observed ones. The second part of the prediction has been verified by professional practices, indicating that this theory is correct, and the predictive method is feasible as well. If conditions permitted, directly measuring the temperatures of the insect’s microhabitats in advance would certainly simplify the analytical process and improve predictive accuracy further.

### 4.3. Comparison of Different Fecundities

There are five articles that have focused on the impacts of different hosts on the development and fecundity of *B. difformis* [21,22,28,34] or *B. impatiens* [27], and a total of 10 vegetable crops and five edible fungi were used in these experiments. The larvae were reared in sandy soils containing decayed beans as food at 26 ± 1 °C [21], in wet filter paper dishes containing tissues of different hosts at 25 ± 1 °C [22,27,28], or in glass cups containing tissues of different hosts at 25 ± 1 °C [34]. The results have been excerpted and are listed below, mostly in Table 5.

*Bradysia difformis* completed its generational development in 20 days, and its fecundity was tested to be (*m* ± *SE*) 72.3 ± 5.1 eggs (*n* = 30; ext. 7–131) per female [21]. Table 5 shows that different host plants and/or fungi significantly influenced the development and fecundity of this sciarid pest. The developmental durations in the immature stage (DDIS) averaged 21.1 d, ranging from the shortest of 16.4 d to the longest of 24.3 d. The survival rates of the immature insects (SRI), with a mean = 44.8%, ranged from the lowest of 12.5% to the highest of 79.5%. Fecundity, with a mean = 70.6, ranged from the smallest of 39.6 to the largest of 106.4 eggs per female.

One of the treatments in our rearing experiments was that larvae were fed with root powders of *P. quinquefolium* at 24.5 °C; under these conditions, *B. impatiens* displayed a DDIS of 21.1 d and an SRI of 52.3% (Table 2). The DDIS was the same as the mean listed in the last line of Table 5, and a little longer than that reported by [21], which should be attributed to the slightly lower rearing temperature in our side. The SRI tested by us was 7.5% higher than the mean in Table 5, clarifying two points, as follows: (1) the effects of rearing larvae in wet sandy soil paper dishes were comparable with those of the methods used by those other authors; and (2) the prevalent larval rearing methods needed improving as the death of the insect was inevitable in its immature stage. The fecundity derived from our observation was 59.2 eggs per female, which is 11.4 eggs less than the mean shown in Table 5, meaning that the sciarid pest had a moderate preference for feeding on American ginseng roots.

### 4.4. Potential Population Size of the Pest and Loss of American Ginseng Caused

With known values of fecundity (*E* = 59.2 eggs per female), the average survival rate of immature insects at 10.0–30.0 °C (*SRI* = 46.4%, or 0.464; Table 2), the female portion (*FP* = 2/3, or 0.667), and the number of generations (*NG* = 4.2 to 5.7) of sciarid pest that would occur in local regions annually, we may estimate the number of females (*NF*) that a parent female and her feminine offspring would reproduce under favorable conditions by the end of *NG* in a year by applying the following exponential growth equation:

$$\begin{array}{ll} \mathrm{NF} & ={\left(E\cdot \mathrm{SRI}\cdot \mathrm{FP}\right)}^{\left(\mathrm{NG}-1\right)}\\ & ={\left(59.2\times 0.464\times 0.667\right)}^{\left(4.2-1\right)}\approx 18.3^{3.2}\approx 10,961(\mathrm{females}),\mathrm{or} \end{array}$$

$$\begin{array}{ll} \mathrm{NF} & ={\left(E\cdot \mathrm{SRI}\cdot \mathrm{FP}\right)}^{\left(\mathrm{NG}-1\right)}\\ & ={\left(59.2\times 0.464\times 0.667\right)}^{\left(5.7-1\right)}\approx 18.3^{4.7}\approx 858,065(\mathrm{females}) \end{array}$$

Here, the expression (*NG* − 1) implies that the equation starts with the 59.2 eggs that a parent female would have laid by the end of Generation 1; the resulting 18.3 F_1_ females would be the base number of Generation 2, and so on. Each of its feminine offspring is supposed to possess the same fecundity as the parent female does.

The sequence extending from one parent female to the number produced by the end of *NG* in a year forms a geometric sequence; therefore, the sum of the first *n* terms of the sequence (*Sn*) can be calculated via the following formula:

$$\begin{array}{ll} Sn & =a_{1}(1-q^{n})/(1-q)\\ & =1(1-18.3^{4.2})/(1-18.3)\approx (-200,582)/(-17.3)\approx 11,594(\mathrm{females}),\mathrm{or} \end{array}$$

$$\begin{array}{ll} Sn & =a_{1}(1-q^{n})/(1-q)\\ & =1(1-18.3^{5.7})/(1-18.3)\approx (-15,702,586)/(-17.3)\approx 907,664(\mathrm{females}) \end{array}$$

Here, the first term *a*_1_ = 1, the common ratio *q* = 18.3, and *n* = *NG* = 4.2 or 5.7.

The total potential population size (*NP*) would be 1.5-fold the value of *Sn*, and with the number of males included, it would range from 17,391 to 1,361,496 adults.

Since the body size of a matured larval *B. impatiens*—about 5 mm long—is similar to that of a matured juvenile or sub-adult (= adult equivalent, AE) of the worm *Enchytraeus buchholzi* (corrected name) [63,64], the average quantity of fresh American ginseng tissues that the latter eats (1.19 mg per AE) [63] is referred to to estimate the fresh mass of American ginseng root eaten away by a parent female *B. impatiens* and all of its offspring in a year. The potential loss of fresh roots would range from 20.7 g (= 1.19 mg × 17,391 ≈ 20,695 mg ≈ 20.7 g) to 1620 g (= 1.19 mg × 1,361,496 ≈ 1,620,180 mg ≈ 1620 g).

American ginseng is a perennial herbaceous plant with precious medicinal roots. It takes four to five years for the plant to grow and ripen. When its germinated seeds were sown in early spring, a one-year-old rootlet weighed 0.70 g by later autumn [65], a two-year-old root averaged 5.85 g [66], and the mean and *SD* of a four-year-old fresh root sample were 23.0 ± 12.7 g (*n* = 173, ext. 2.8–64.1). Under these circumstance, the potential loss of fresh roots mentioned above would be extremely severe if the sciarid pest density were as low as just one female per seedling in the field in early spring; the parent female and her offspring would eat away a one-year-old rootlet and/or a two-year-old root entirely within a year. The threat of the pest ruining seedlings (Figure 15B) exists all the time as the pest’s population is growing, though its progenies may disperse to adjacent seedlings, or its population growth may change from exponential to logistic, just as in the example concerning the worm *Enchytraeus buchholzi* [67].

## 5. Conclusions

To protect local American ginseng production from subterranean pest injuries, we have studied herein the sciarid fly as regards its taxonomic position, the relationship between its development and temperature, and its reproductive potential. Based on characteristic microphotographs taken of specimens, and comparisons of our observations with specific descriptions and free-hand drawings published previously, our morphotaxonomic identification has confirmed that the sciarid fly is *Bradysia impatiens* (Johannsen); furthermore, our experiment via DNA barcoding has endowed this identification with strong evidence. The literature review shows that this sciarid pest attacks vegetable crops, edible mushrooms, ornamental plants, and several types of herbage in many parts of China.

Following the principle of “Thermal Sums”, we conducted serial experiments rearing the species from egg to adult at eight temperatures, calculated the developmental threshold temperature (*C*) and thermal constant (*K*) of the insect in its egg, larval, pupal and generational stages, and then constructed four reciprocal models for its developmental duration (*N*) with temperature (*T*), *N* = *K*/(*T − C*). With *K, C* and *SE* all known, the model of generational development is *N_generation_* = (374.9 ± 8.15)/[*T* − (6.6 ± 0.29)]. Let the annual effective accumulated temperatures in the soil at a 10 cm depth be divided by *K* (374.9 d°C); we can thus foresee that the sciarid pest may occur at a rate of 4.2 to 5.7 generations a year in local areas. The prediction that “the sciarid pest may occur for 4.2 generations a year in Zhakoushi Village” has been verified by practical investigations.

The fecundity was tested to be 59.2 eggs per female under favorable conditions; the average survival rate of the immature insect was 46.4% in the range from 10.0 to 30.0 °C, and the sex ratio of female to male was 2:1, or put differently, the female portion was 0.667. Based on these data, we estimate that a parent female would reproduce 18.3 filial females by the end of Generation 1, and the number of her offspring could reach 11,594–907,664 females and/or 17,391–1,361,496 adults if she and her feminine offspring reproduced for 4.2–5.7 generations a year. Correspondingly, a total of 20.7–1620 g of American ginseng roots would be eaten away, with more tissues contaminated and wasted.

Having made the identity of *B. impatiens* clear, along with information about its physiological time and reproductive potential, we may understand more about its occurrence, developmental and reproductive tendencies, which will help us to protect American ginseng production from damage and ruin caused by this sciarid fly more efficiently.

## Figures and Tables

**Figure 1:**
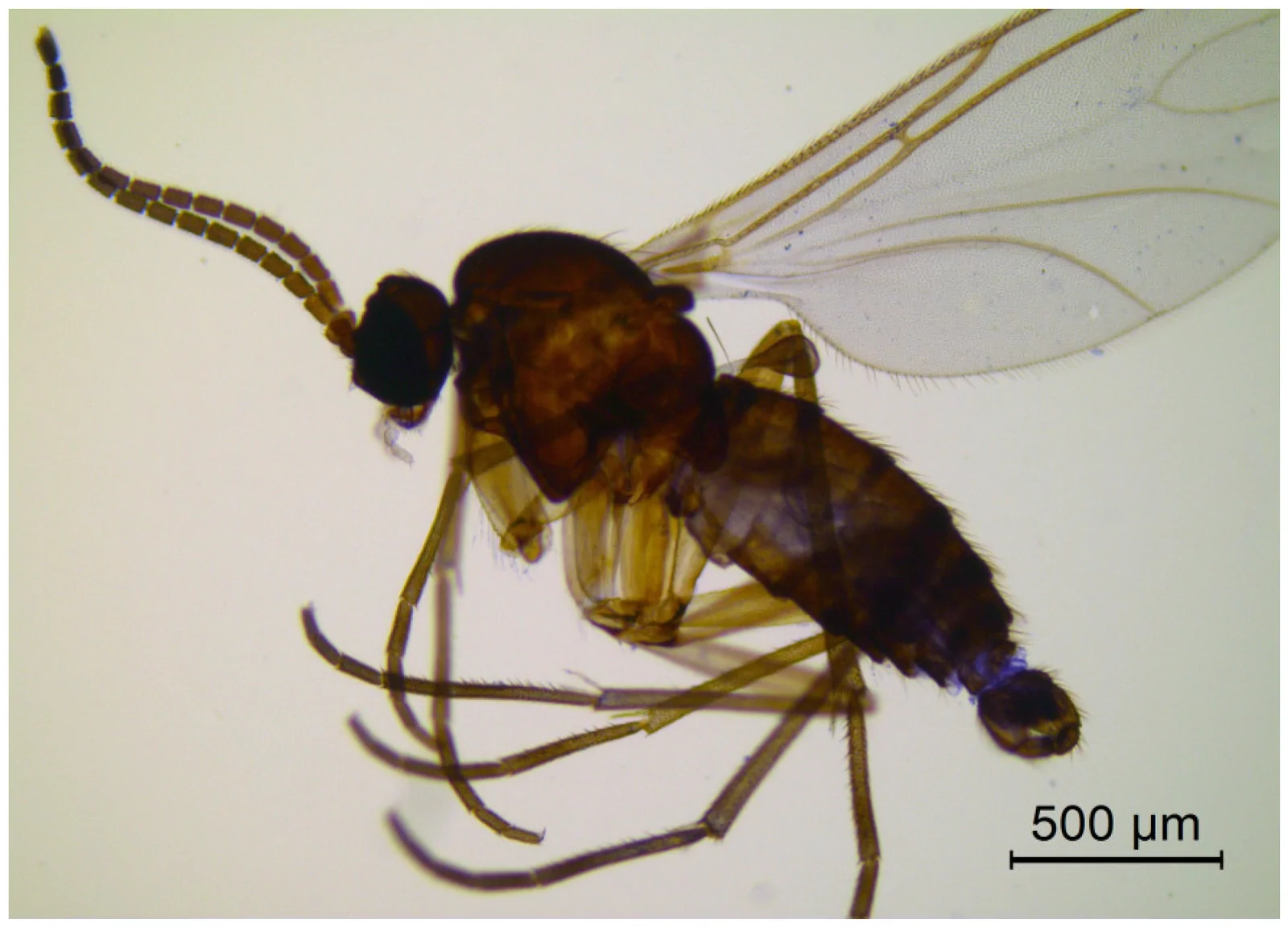
Body form of an adult male mounted whole on a glass slide, lateral view.

**Figure 2:**
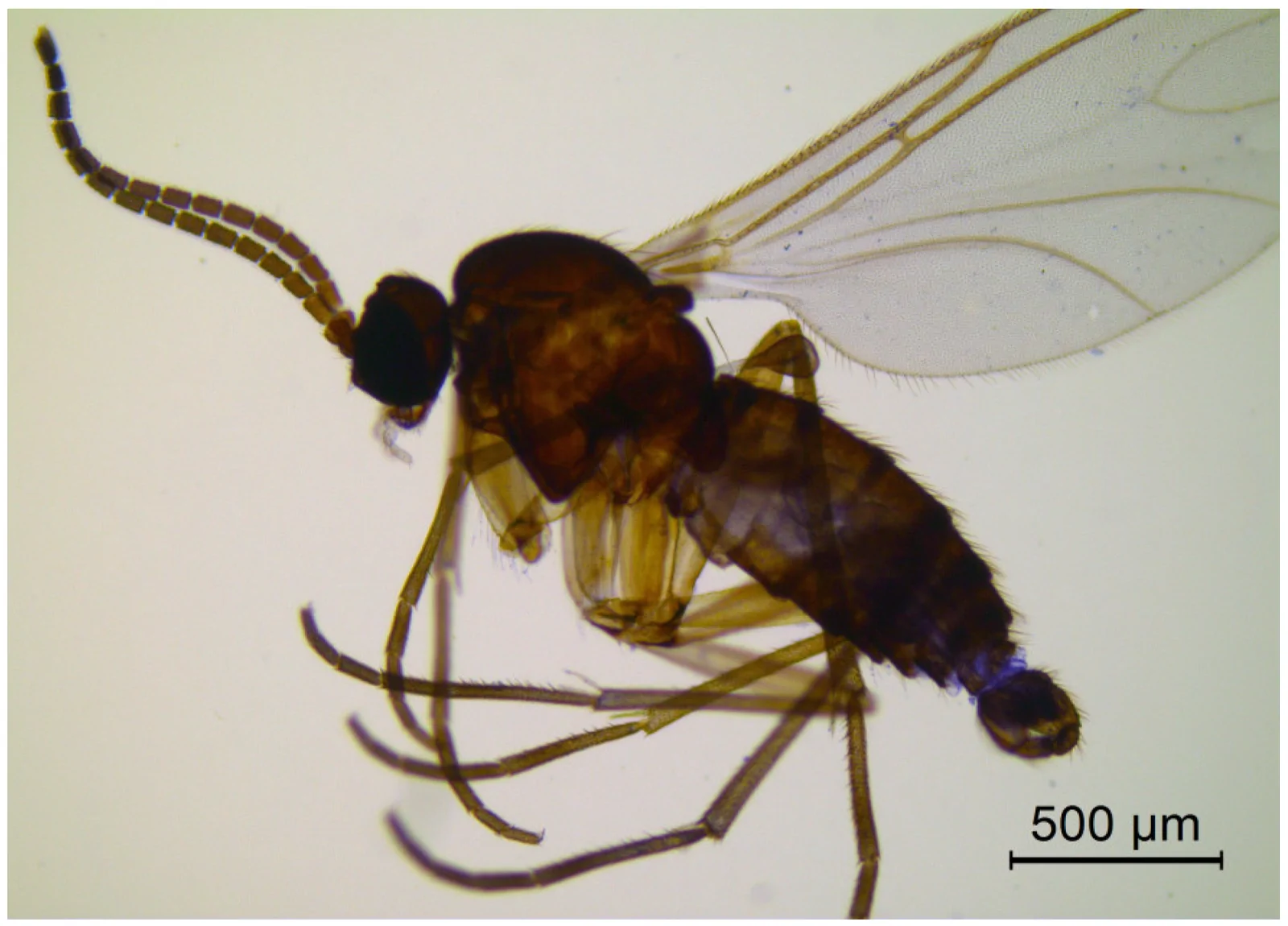
Body form of an adult female with her eggs just laid, lateral view, observed alive.

**Figure 3:**
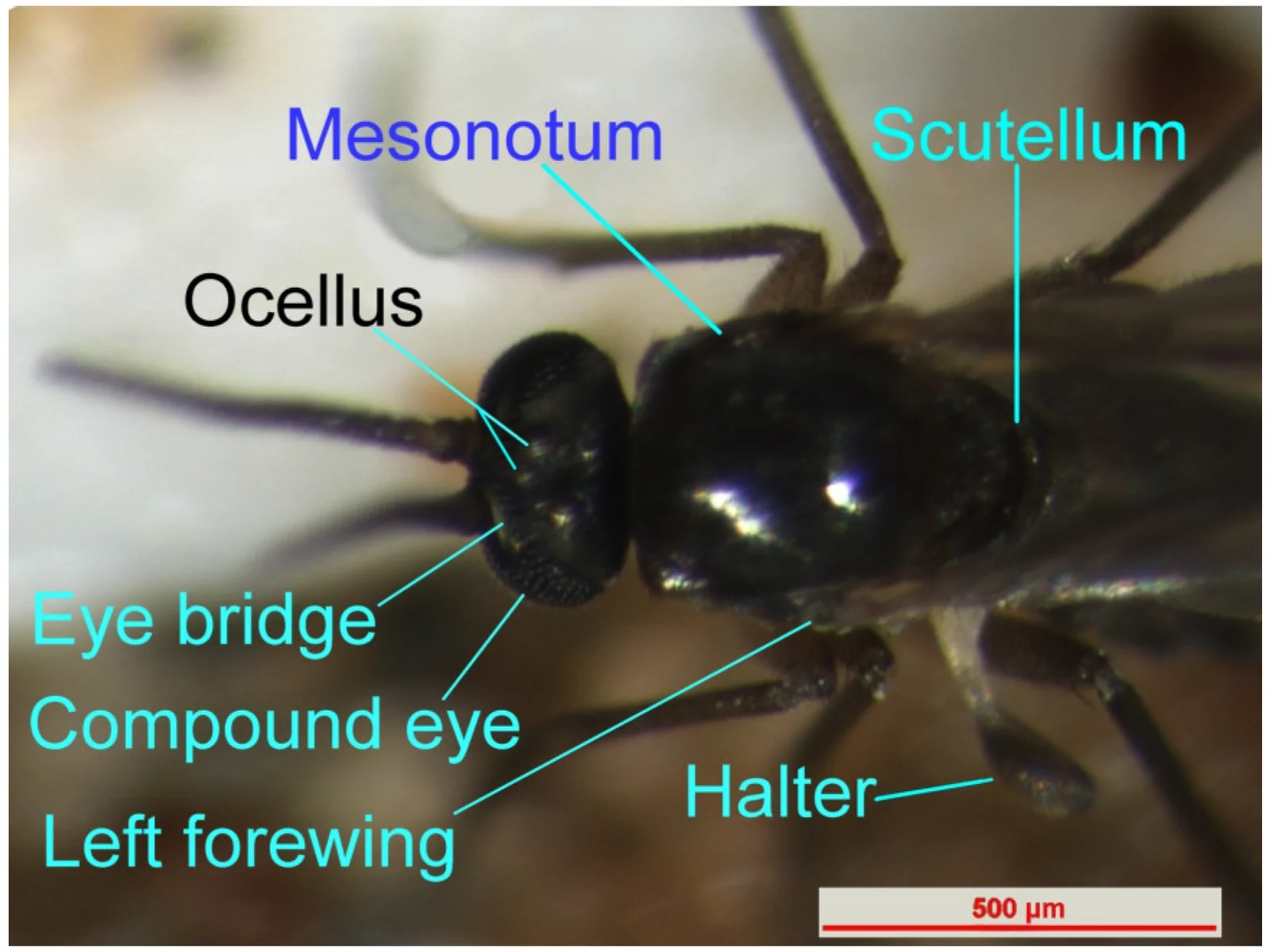
Head and thorax with appendages such as antennae, legs, wings and halteres; female, dorsal view, observed alive.

**Figure 4:**
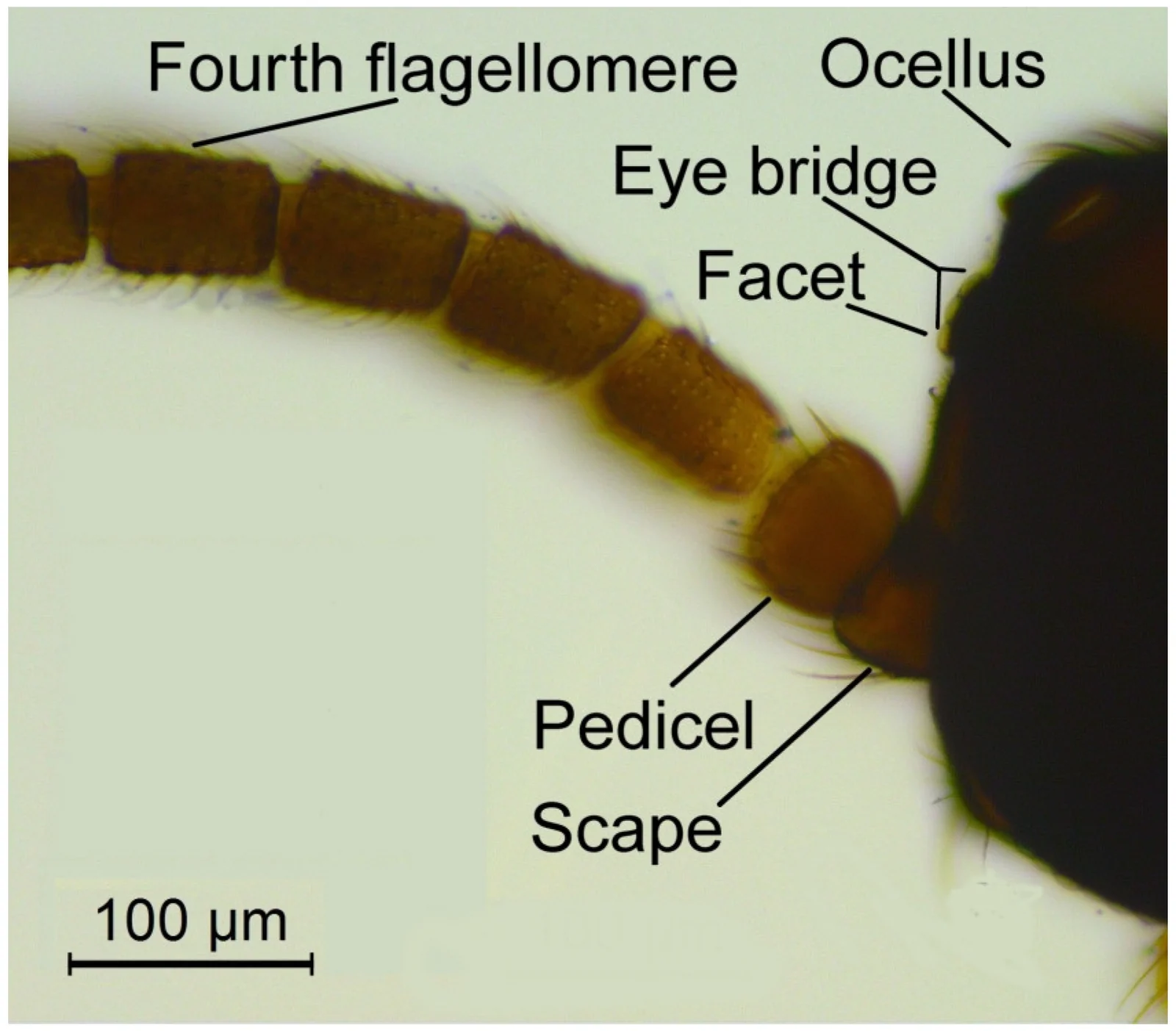
Head and antenna in part, lateral view, showing the fourth flagellomere, ocellus, eye bridge, facet, etc.

**Figure 5:**
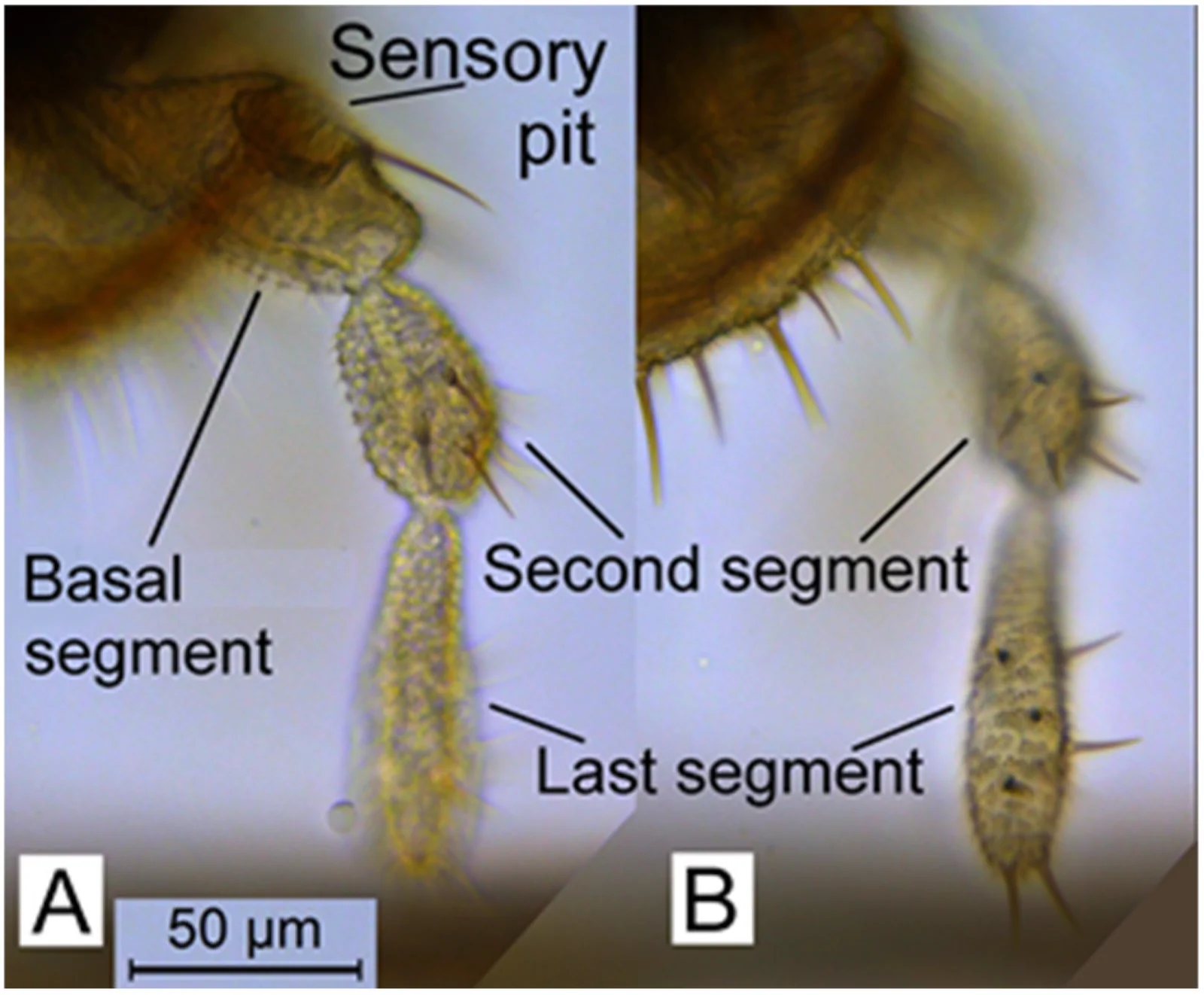
Maxillary palpus. (**A**,**B**) Microphotographs of the palpus, showing sensory pit, basal, second and last segments, which are in different focus planes.

**Figure 6:**
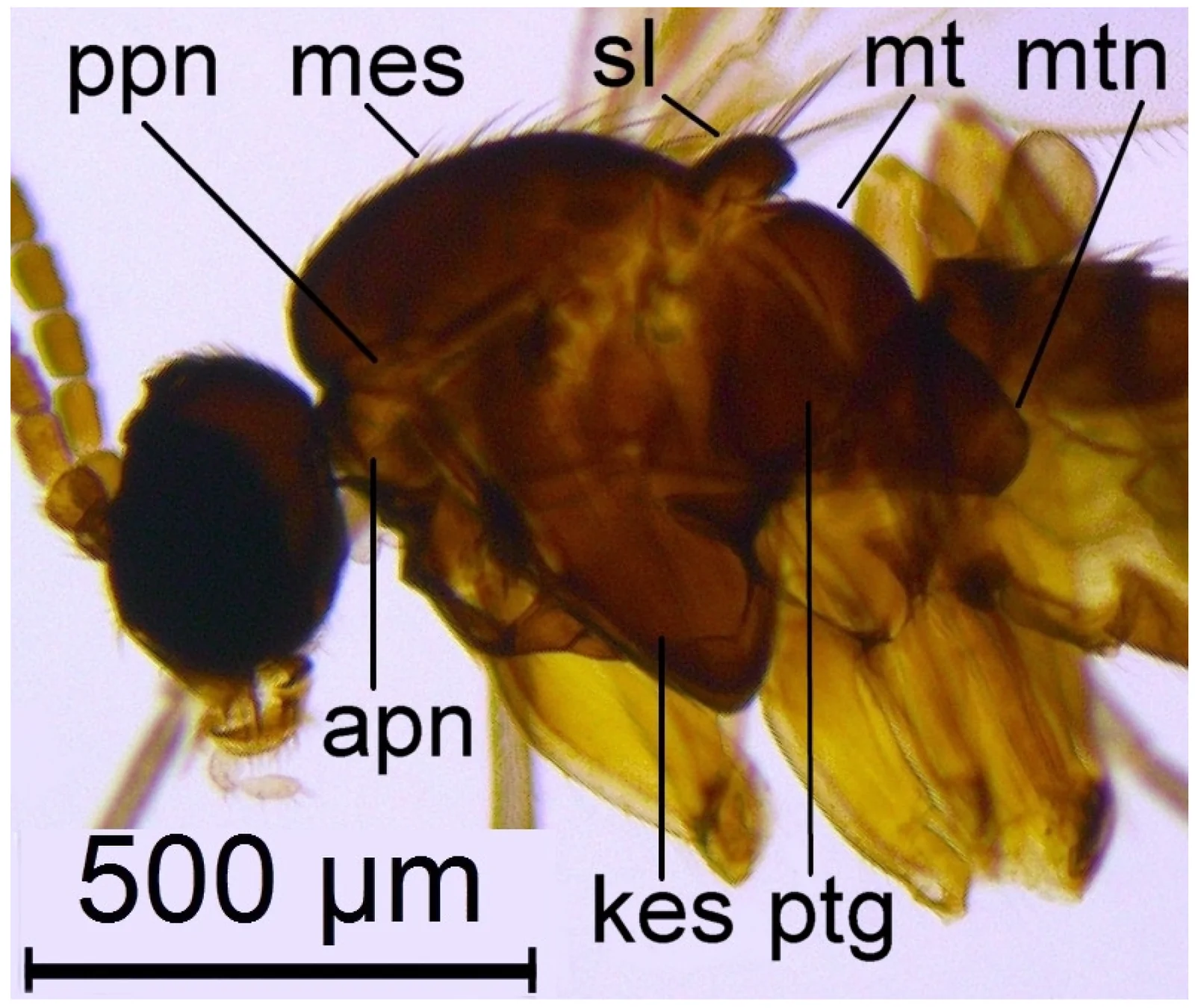
Thorax of male on a glass slide, lateral view. apn, antepronotum; kes, katepisternum; mes, mesonotum; mt, mediotergite; mtn, metanotum; ppn, postpronotum; ptg, laterotergite; sl, scutellum. Terms from Mohrig et al. (2013) [4].

**Figure 7:**
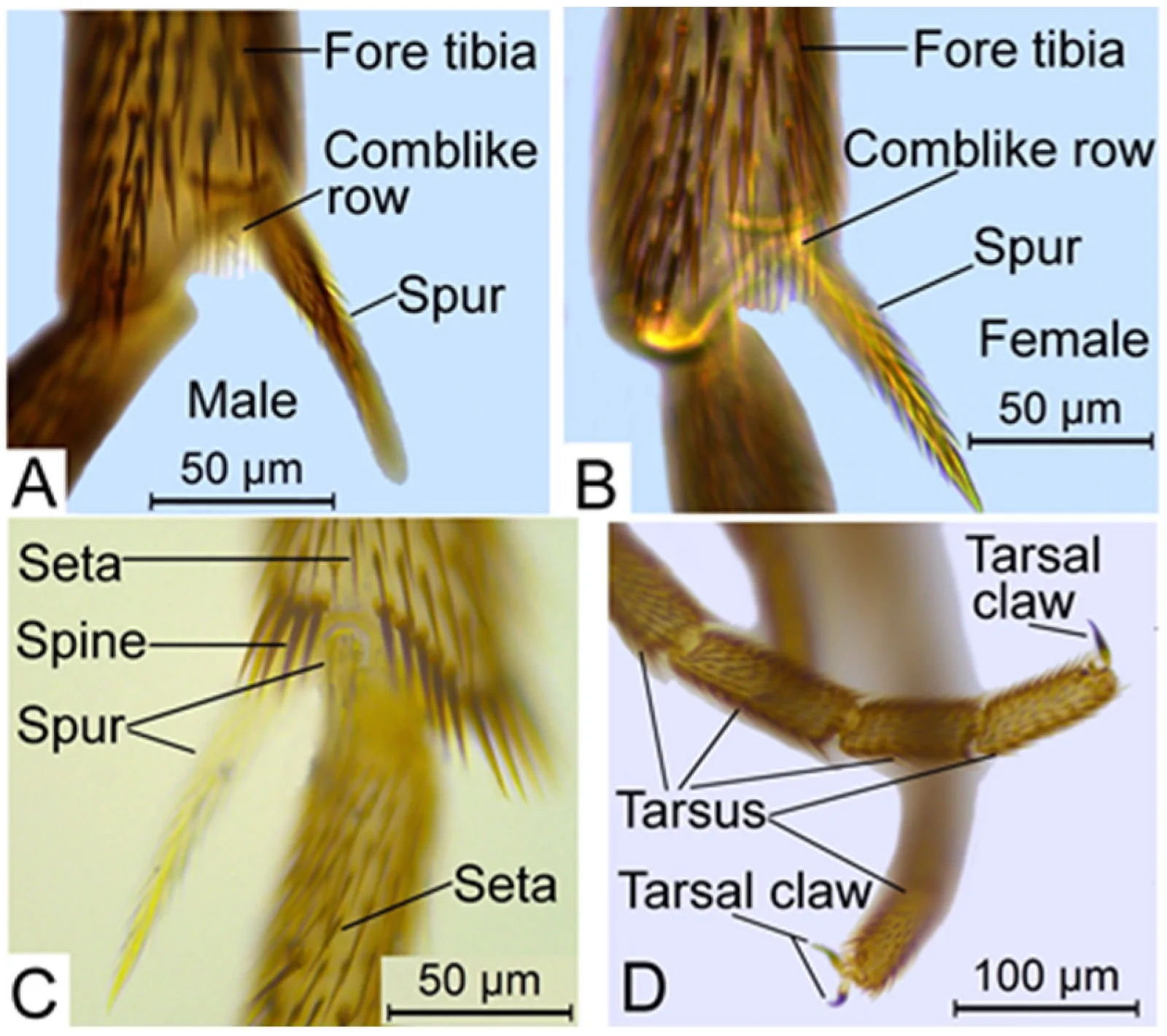
Distal end of tibia, lateral view. (**A**) Comb-like row of male fore tibia. (**B**) Comb-like row of female fore tibia. (**C**) Two spurs and many apical spines around female mid and hind tibiae. (**D**) Tarsus and tarsal claw.

**Figure 8:**
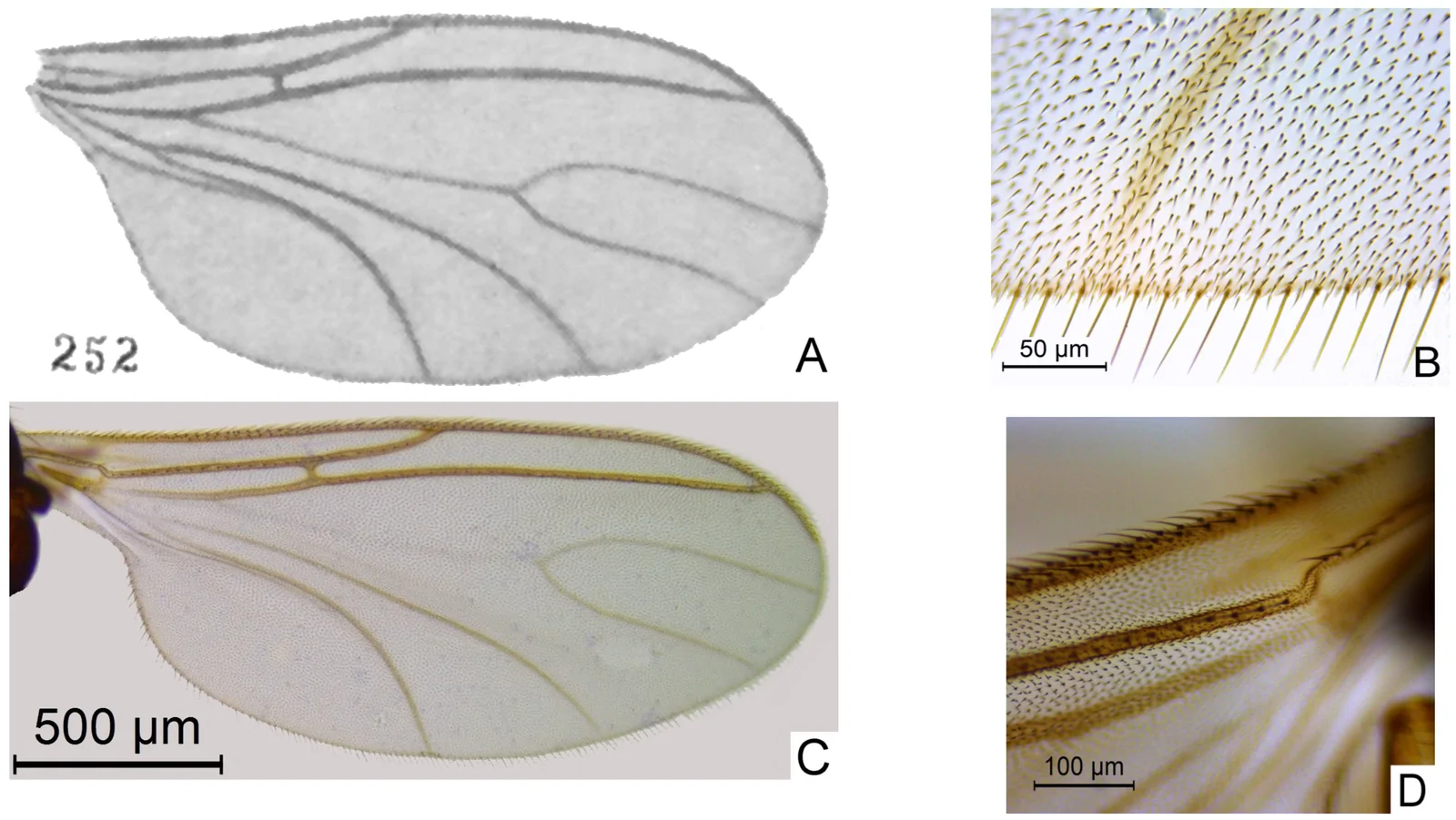
Wing and venation, male, dorsal view. (**A**) A drawing of the male forewing, reprinted from Ref. [1]. (**B**) An area of wing membrane, covered with dense microtrichiae, and setae along the wing edge that are relatively longer. (**C**) Forewing of male. (**D**) Basal parts of costa and radius, showing the arrangement of macrotrichiae.

**Figure 9:**
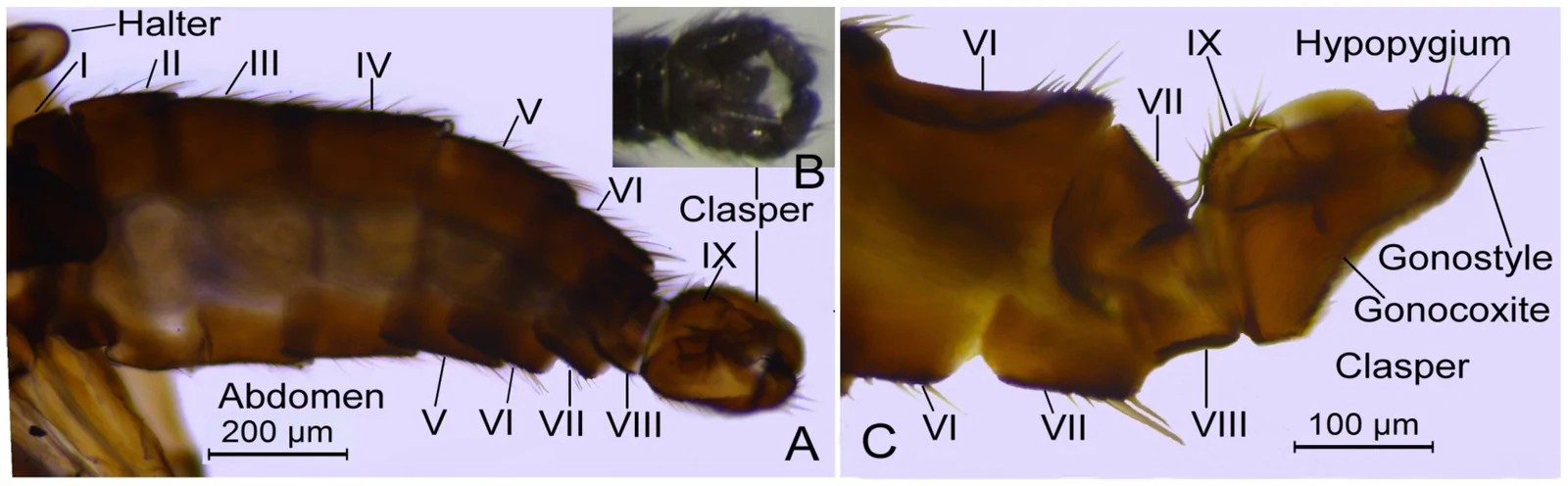
Male abdomen with hypopygium. (**A**) Abdomen on a glass slide, lateral view, consisting of nine segments, and the appendages on the last segment are specialized into a pair of gonocoxites and gonostyles that form a hypopygium or a pair of claspers. (**B**) Segments VII and VIII and the hypopygium, observed alive, dorsal view, showing a color that is fuscous or black. (**C**) Segments VI–VIII and the hypopygium, lateral view.

**Figure 10:**
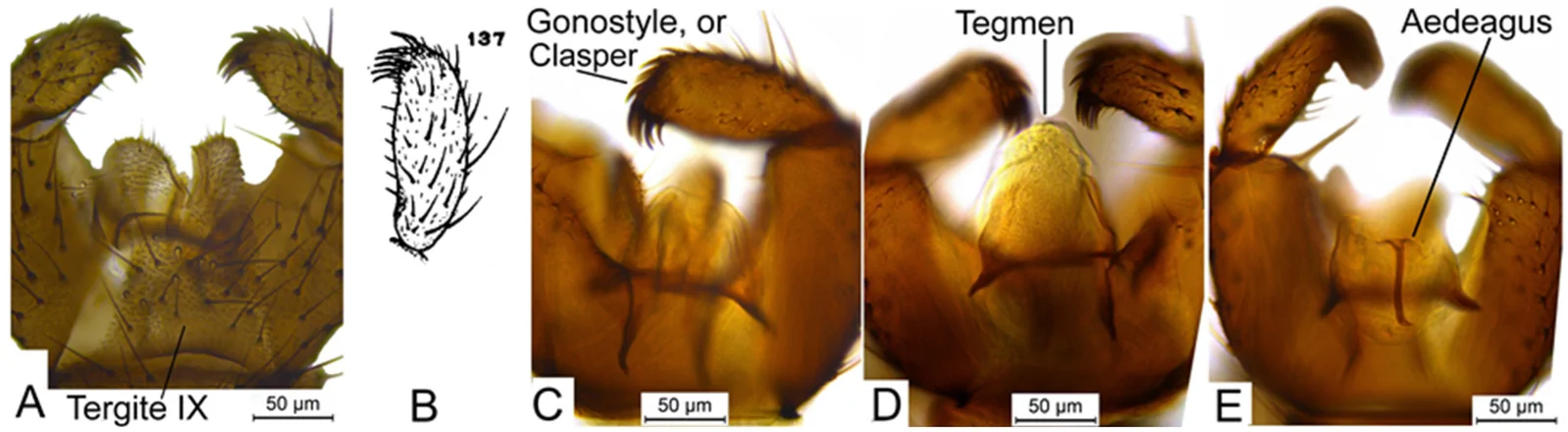
Tergite IX and hypopygium. (**A**) Tergite IX of male, dorsal view, showing its position and connection with gonocoxites laterally, and a pair of cushion-like cerci with setae (also see Figure 10C). (**B**) A drawing of the clasper (= gonostyle), reprinted from Ref. [1]. (**C**) Dorsal view, showing the gonostyle with one apical tooth and seven subapical spines directed ventromedially. (**D**) Ventral view, showing tegmen. (**E**) Ventral view, showing aedeagus. (**C**–**E**) are all in different focal planes.

**Figure 11:**
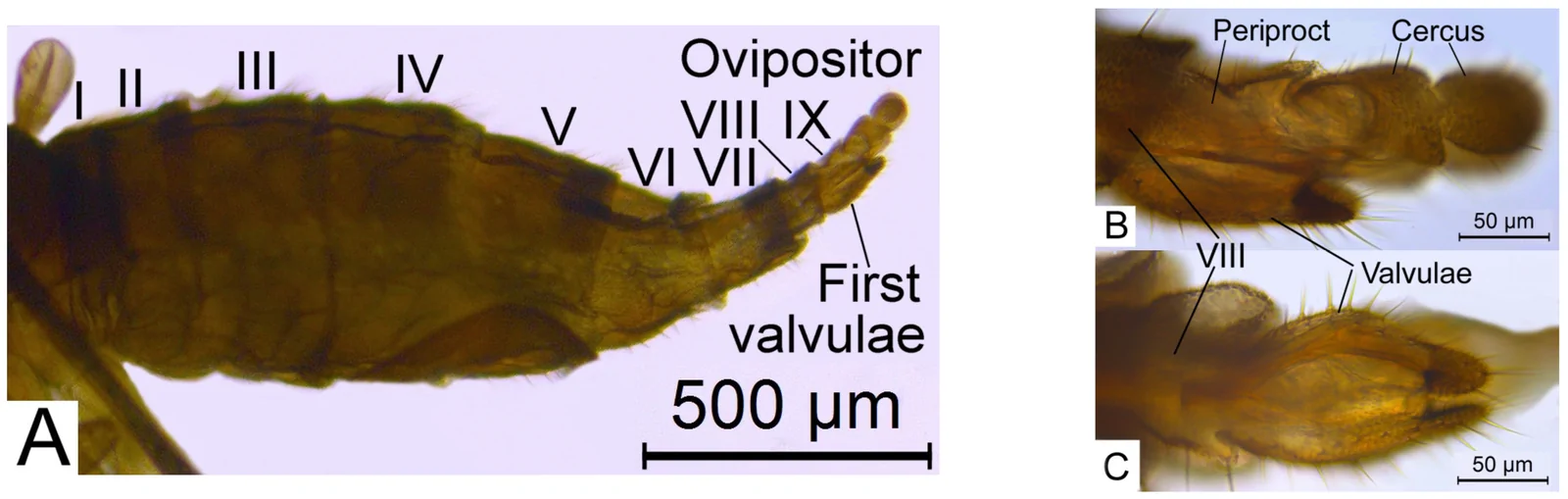
Female abdomen with ovipositor on glass slides. (**A**) Abdomen, lateral view, consisting of nine segments, the last of which is combined with the valvulae to form the ovipositor. (**B**) Segment VIII and ovipositor, lateral view, showing valvulae, periproct and cercus. (**C**) Segment VIII and ovipositor, ventral view, showing that the valvulae have a curved stem and cone-like tip, all with setae.

**Figure 12:**
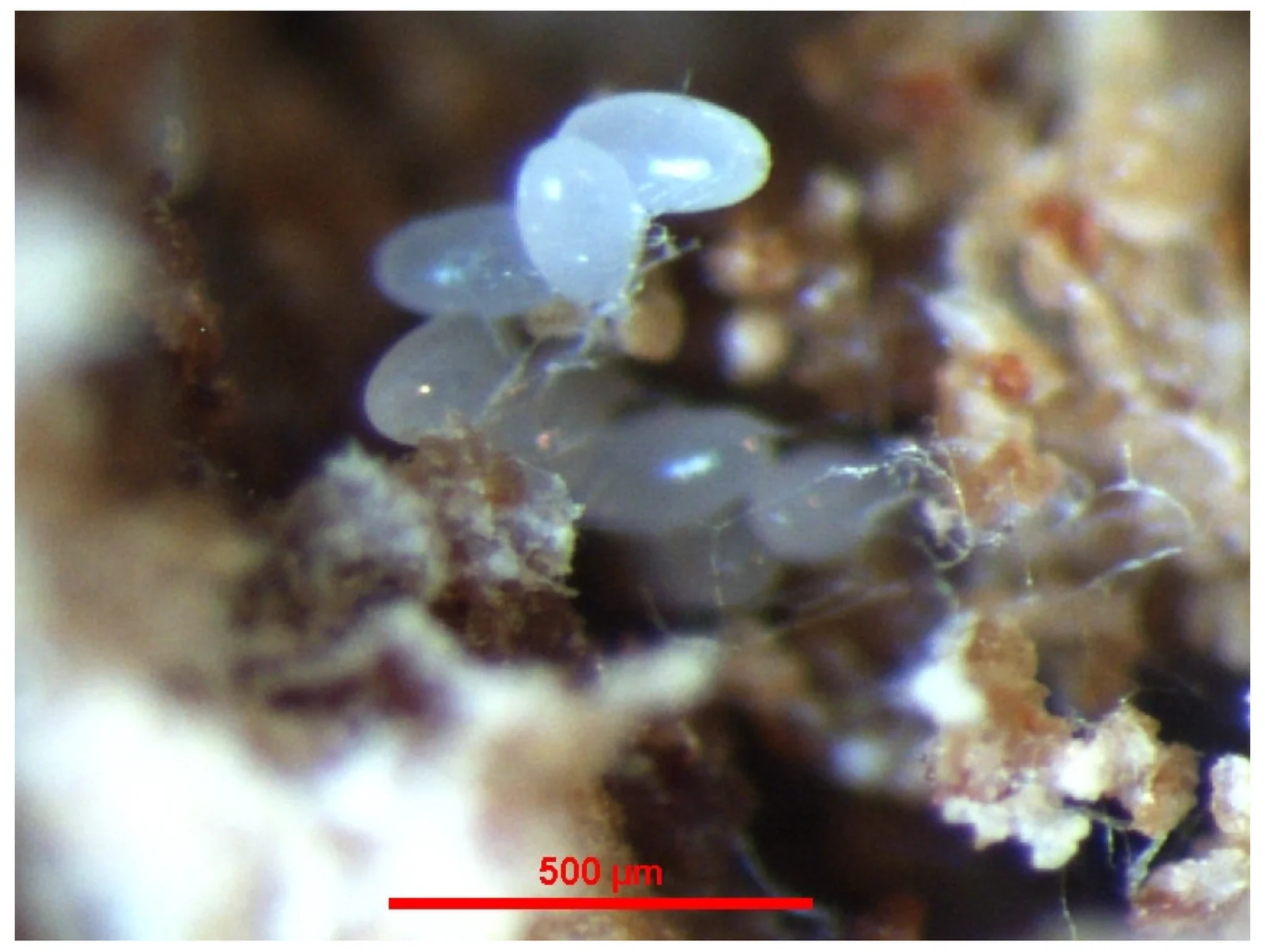
Eggs, laid inside a root of an injured American ginseng seedling, lateral view.

**Figure 13:**
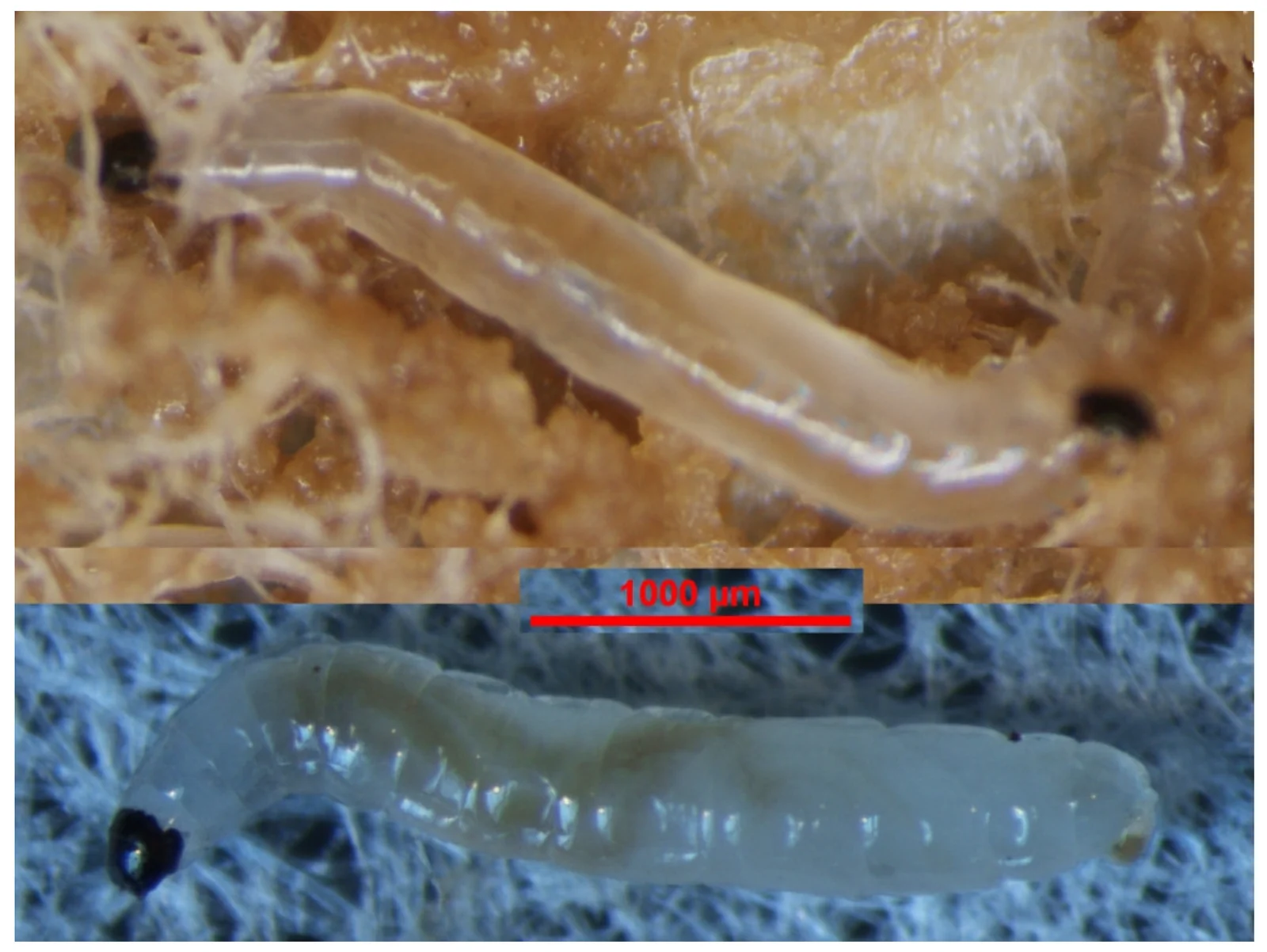
Larva, apoda- and eucephalous-shaped, dorsal view.

**Figure 14:**
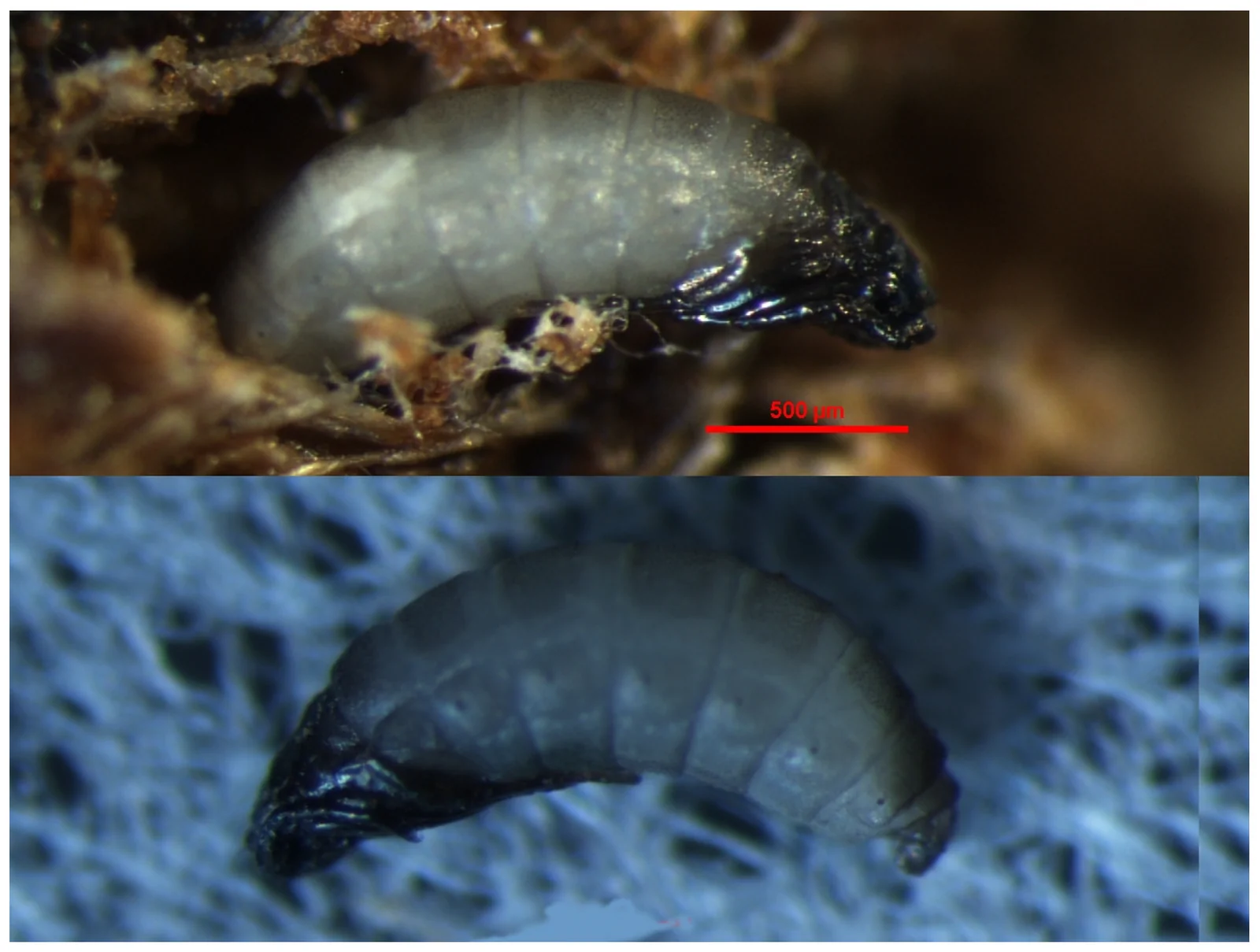
Pupa, obtect, crescent-shaped, lateral view.

**Figure 15:**
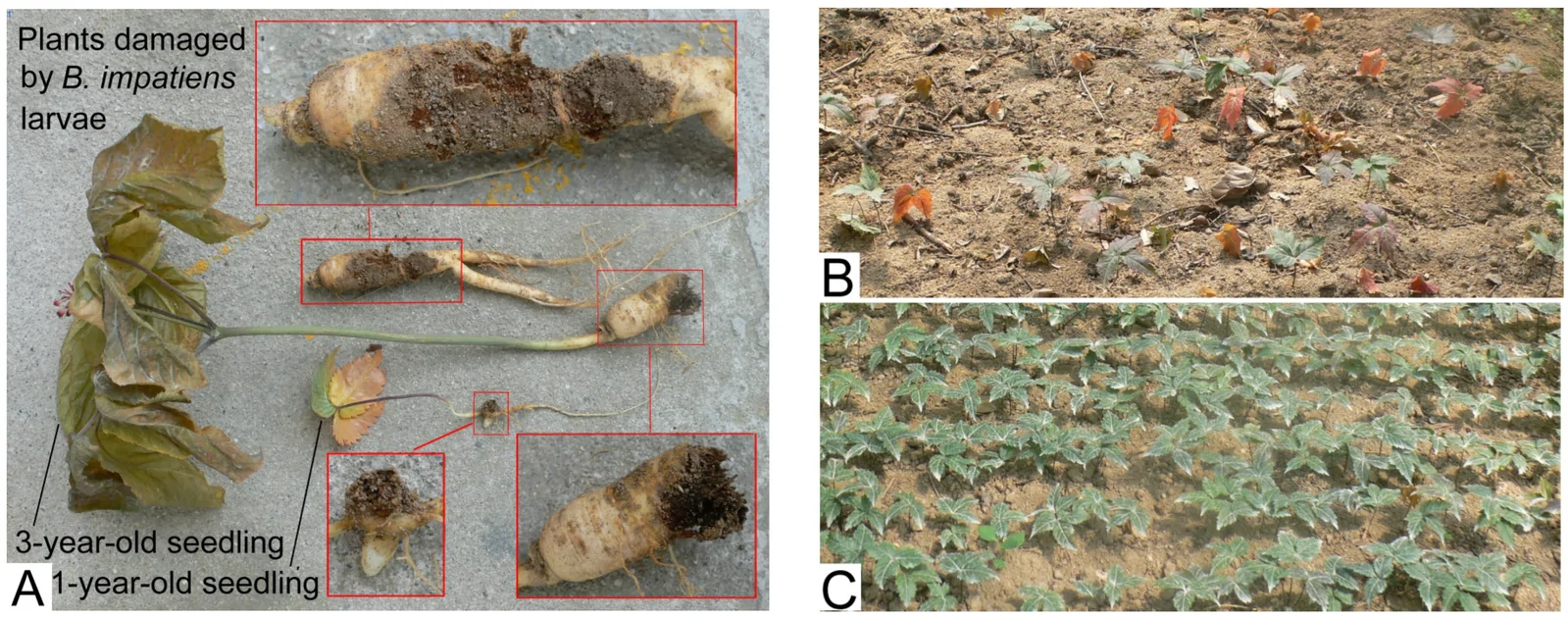
American ginseng seedlings. (**A**) Both one-year-old and three-year-old seedlings were injured by *Bradysia impatiens* larvae. (**B**) An American ginseng plot was ruined by the sciarid pest, leaving only a few seedlings turning yellow or brown and dying soon. (**C**) The one-year-old seedlings in protected plots were growing vigorously (Zaomulan Village, Liuba; August 2013).

**Figure 16:**
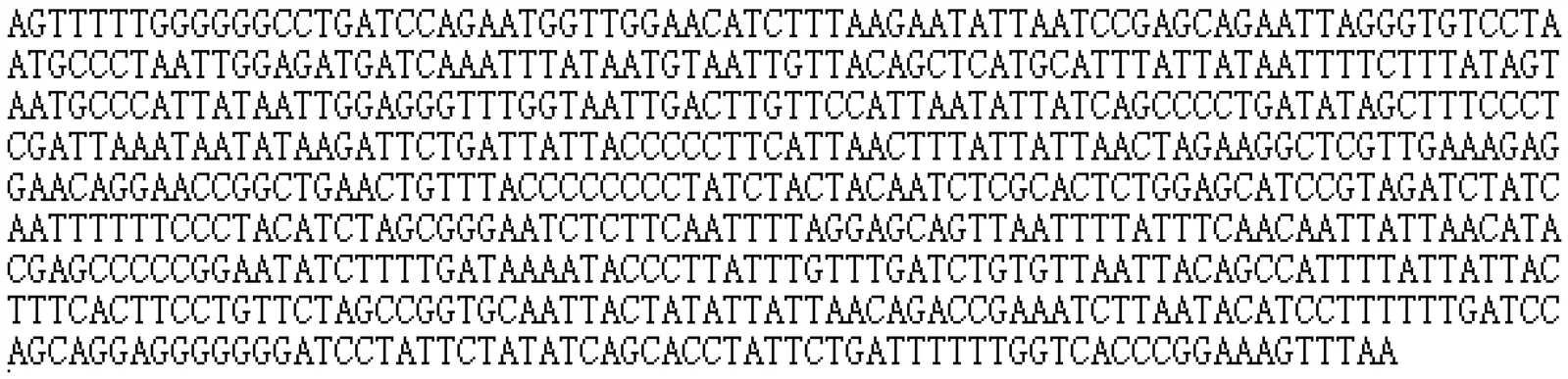
Schematic diagram of the DNA sequence in the mtCOI gene of adult *Bradysia impatiens*. Those of larva and pupa are slightly different, and thus omitted here to save space.

**Figure 17:**
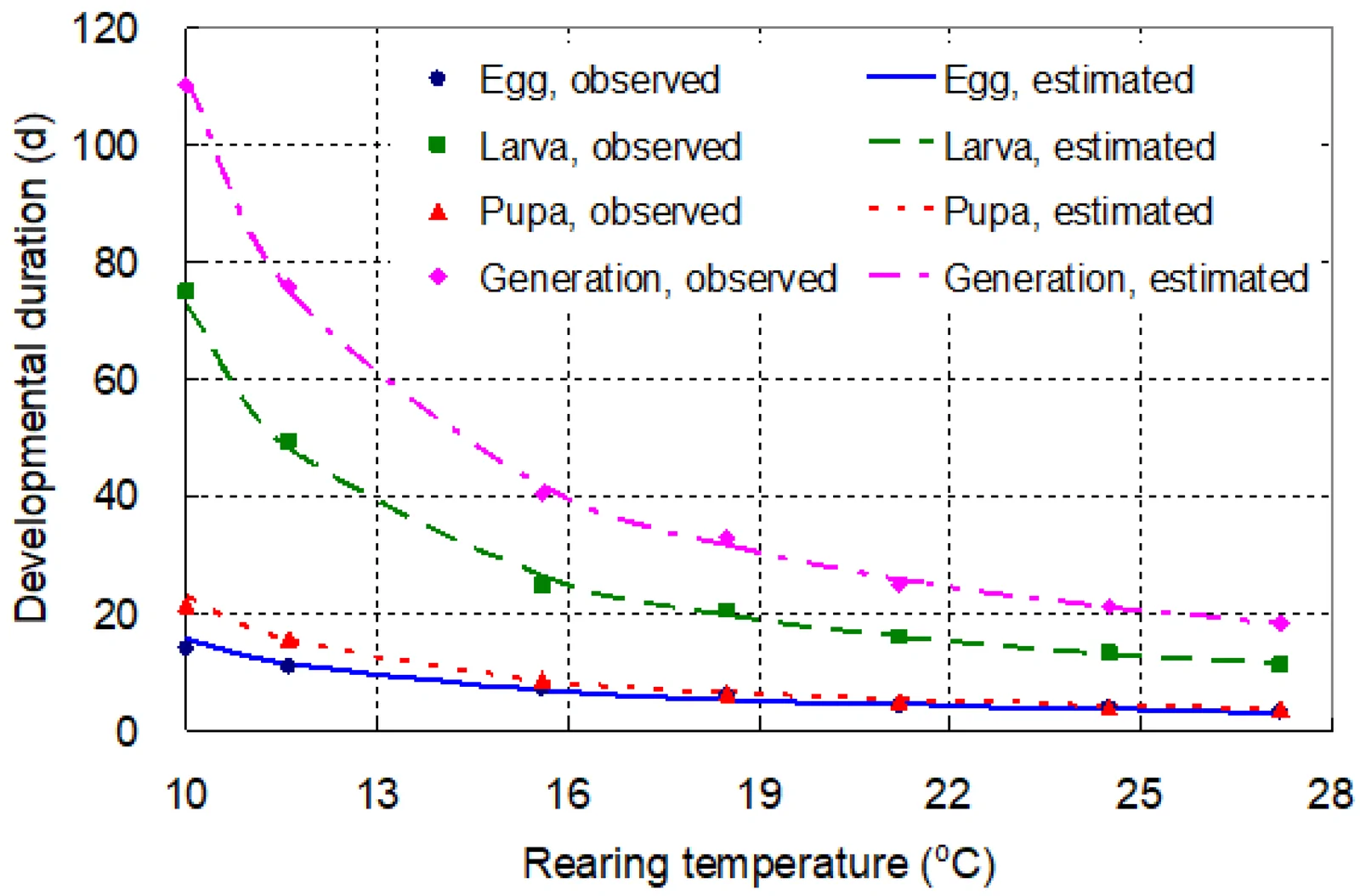
Curves of developmental durations of *Bradysia impatiens* in its four life stages at seven rearing temperatures (the duration of an immature stage is equal to that of a generation, though it does not equate to a full one including pre-oviposition stage).

**Figure 18:**
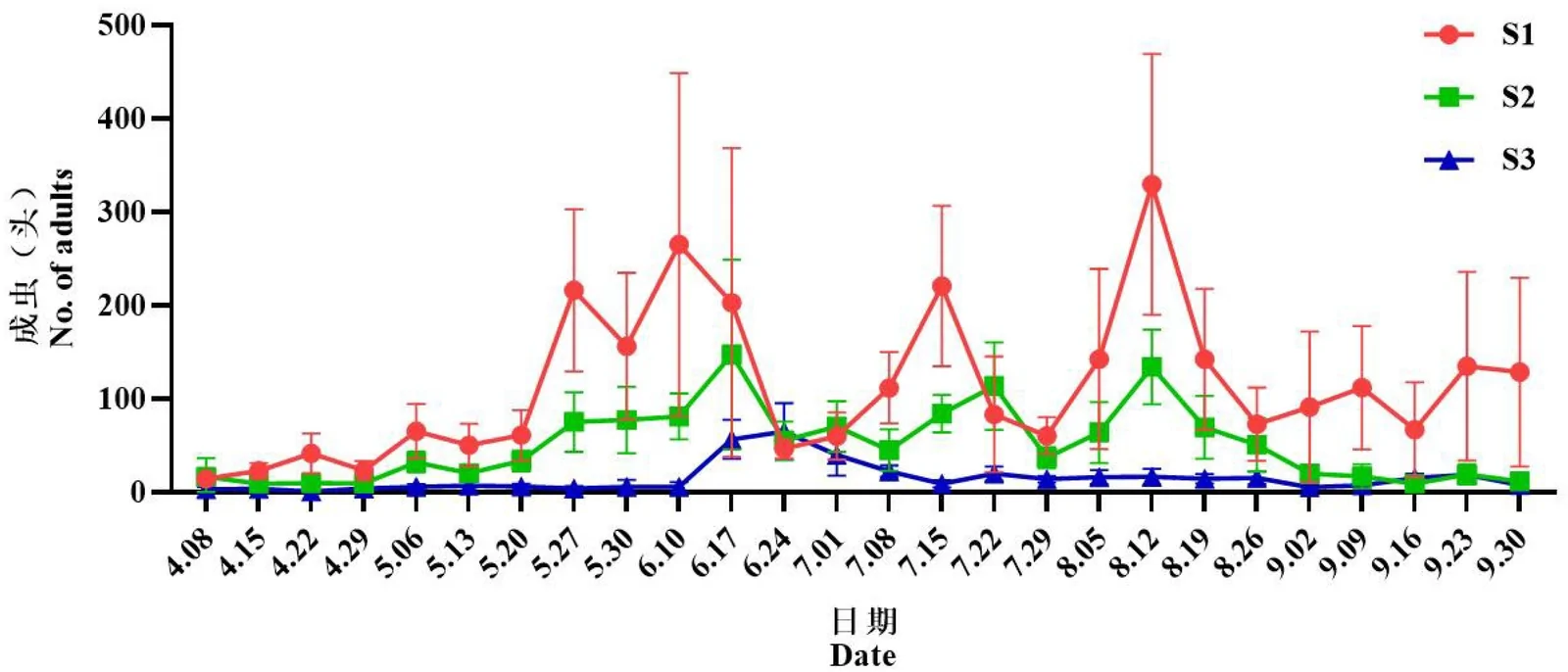
Population dynamics of adult *Bradysia impatiens* at S1, S2 and S3 sample sites in 2022, reprinted with permission from Ref. [37], Copyright 2023 Hao Zhang and the Northwest A&F University, China. S1 was set in a plot of two-year-old American ginseng seedlings, S2 in a plot of four-year-old American ginseng plants, and both were located in Zhakoushi Village (alt. 1653 m). S3 was set in a plot of four-year-old American ginseng plants in Taoyuanpu Village (alt. 1109 m).

**Figure 19:**
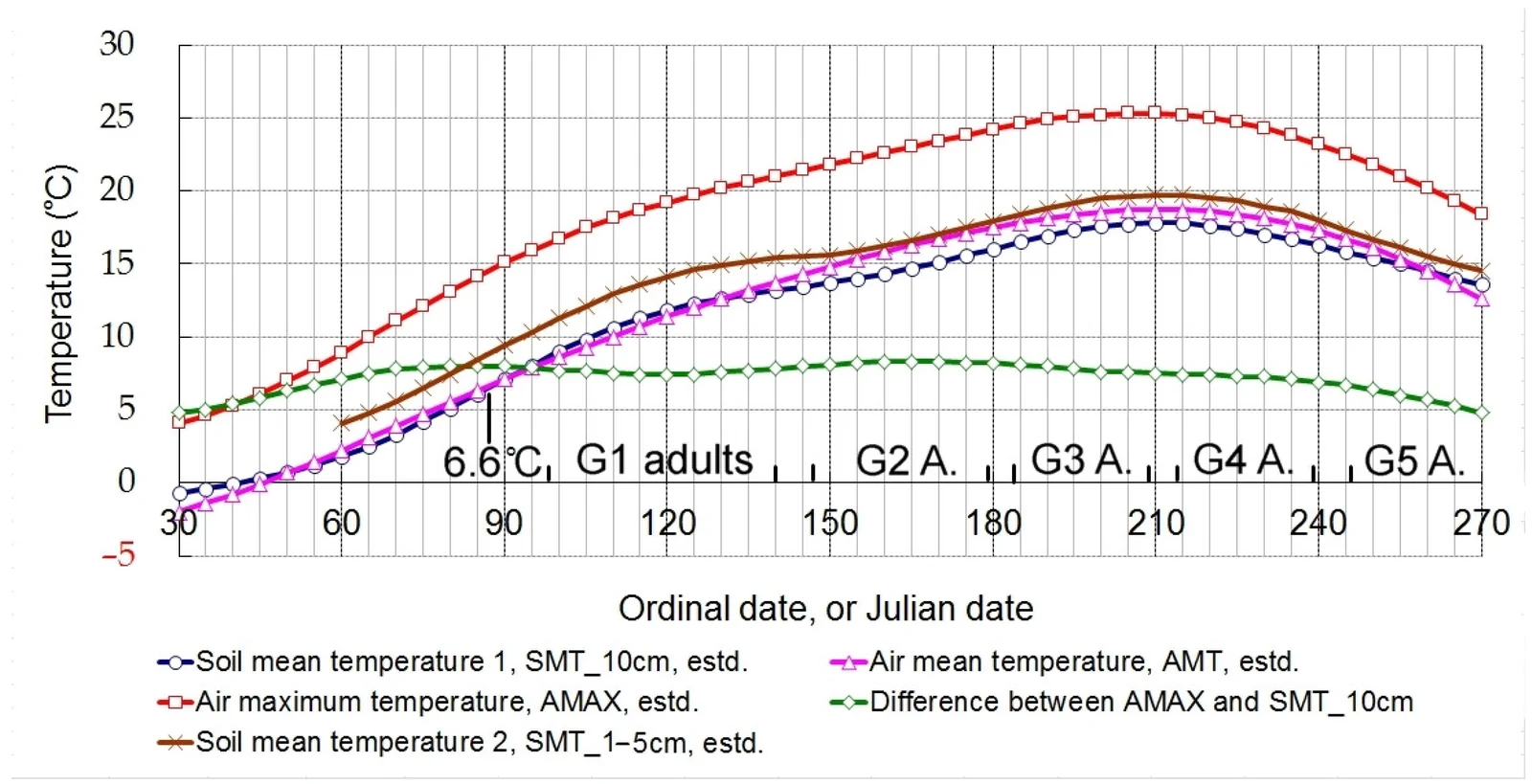
Periodical temperature curves (in part) within plastic greenhouses for American ginseng growth in Zhakoushi Village (alt. 1722 m) in 2012–2014. Air temperatures were measured at 15 cm aboveground around canopies of two-year-old seedlings. Soil mean temperature 1, or SMT_10 cm, at 10 cm underground near host roots, adapted from Ref. [61]. Soil mean temperature 2, or SMT_1–5 cm, adjusted to 1–5 cm depth (A recombined version).

**Table 1:** Identification, host plants and edible fungi, and infested areas of *Bradysia impatiens* in China, including its synonym *B. difformis.*

Species	Identification ^A^	Host Plants and Fungi	Infested Areas	Reference
*B. difformis*	Morphotaxonomy	Nine edible mushrooms ^B^	Yunnan (eight counties)	[12]
*B. difformis*	Morphotaxonomy	Eight edible fungi	Yunnan (eight counties)	[13]
*B. difformis*	Morphotaxonomy	*Kalanchoe bolssfeldiana*	Guangdong	[14]
*B. difformis*	Morph. + DNA barc.	*Auricularia cornea*	Jiangsu (Nanjing)	[15]
*B. difformis*	Morph. + DNA barc.	Five edible mushrooms ^C^	Guangxi (Nanning)	[16]
*B. difformis*	Morph. + DNA barc.	*Pleurotus ostreatus*	Guangxi (Nanning)	[17]
*B. difformis*	Morph. + DNA barc.	*Pleurotus ostreatus*	Zhejiang (Lin’an)	[18]
*B. difformis*	Morph. + DNA barc.	*Boletus edulis*, *Boletus* sp.	Yunnan (Kunming, Lijiang)	[19]
*B. difformis*	Morph. + DNA barc.	*Pleurotus ostreatus*	Guangxi (Nanning)	[20]
*B. difformis*	Morphotaxonomy	*Pleurotus ostreatus*	Zhejiang (Lin’an)	[21]
*B. difformis*	Morphotaxonomy	Four vegetables ^D^	Gansu (Gangu)	[22]
*B. difformis*	Morphotaxonomy	*Allium tuberosum*	Gansu (Gangu)	[23]
*B. difformis*	Morph. + DNA barc.	*Phalaenopsis* orchid	Guangdong	[24]
*B. difformis*	Morph. + DNA barc.	*Pleurotus ostreatus*	Guangxi (Nanning)	[25]
*B. impatiens*	Morphotaxonomy	*Pleurotus ostreatus*	Zhejiang (Lin’an)	[26]
*B. impatiens*	Morphotaxonomy	Four edible fungi ^E^	Zhejiang (Lin’an)	[27]
*B. difformis*	Morphotaxonomy	Five vegetables ^F^	Gansu (Gangu)	[28]
*B. difformis*	Morphotaxonomy	*Allium tuberosum*	Liaoning (Shenyang)	[29]
*B. difformis*	Morphotaxonomy	*Allium tuberosum*	Shandong	[30]
*B. impatiens*	Morph. + DNA barc.	*Pleurotus pulmonarius*	Jiangsu (Guannan, Gaochun) Zhejiang (Chun’an, Jiangshan)	[31]
*B. impatiens*	Morphotaxonomy	*Lactarius* spp. ^G^	Yunnan	[32]
*B. impatiens*	Morphotaxonomy	Five herbages ^H^	Gansu	[33]
*B. difformis*	Morphotaxonomy	Five fungi & one potato ^I^	Yunnan (Mengzi)	[34]
*B. impatiens*	Morph. + DNA barc.	*Anoectochilus roxburghii*	Guangdong (Huizhou)	[35]
*B. impatiens*	Morph. + DNA barc.	Three edible fungi ^J^	Gansu (Longxi, Dingxi)	[36]
*B. impatiens*	Morph. + DNA barc.	*Panax quinquefolium*	Shaanxi (Liuba)	[37]
*B. impatiens*	Morphotaxonomy	Three edible fungi ^K^	Fujian (Fuzhou)	[38]
*B. impatiens*	Morph. + DNA barc.	*Auricularia* spp. ^L^	Shandong, Jiangsu, Fujian, Guizhou, Sichuan	[39]

^A^ Morph. + DNA barc.: Morphotaxonomy plus mitochondrial COI DNA barcoding technology. ^B^ Nine edible mushrooms: *Pleurotus ostreatus*, *Lentinus edodes*, *Agaricus bisporus*, *Agrocybe aegerita*, *Agrocybe chaxingu*, *Coprinus comalus*, *Flammulina velutipes*, *Stmpharia rugosoannulata*, *Pleurotus cirinopileatus*. ^C^ Five edible mushrooms: *Pleurotus ostreatus*, *Lentinula edodes*, *Flammulina velutiper*, *Coprinus comatus*, *Agaricus bisporus*. ^D^ Four vegetables: Chinese chive, *Allium tuberosum*; spring onion, *A. fistulosum*; garlic (bulb and stem), *A. sativum*; lily, *Lilium davidii* var. *unicolor*. ^E^ Four edible fungi: shiitake, *Lentinus edodes*; oyster mushroom, *Pleurotus ostreatus*; *Agrocybe cylindracea*, *Agaricus subrufescens*. ^F^ Five vegetables: leek, *Allium tuberosum*; fava beans, *Vicia faba*; lettuce, *Lactuca sativa*; Chinese cabbage, *Brassica oleracea*; cabbage, *Brassica oleracea* var. *capitata rubra*. ^G^ *Lactarius* spp.: *Lactarius deliciosus*, *L. hatsudake*, *L. vividus*. ^H^ Five herbages: *Medicago sativa*, *Astragalus adsurgens*, *Oxytropis ochrocephala*, *O. kansuensis*, *O. glabra*. ^I^ Five fungi and one potato: *Lentinus edodes*, *Pleurotus ostreatus*, *Allium tuberosum*, *Dioscorea opposite*, *Flammulina velutipes*, *Solanum tuberosum*. ^J^ Three edible fungi: *Lentinus edodes*, *Stmpharia rugosoannulata*, *Pleurotus ostreatus*. ^K^ Three edible fungi: *Hspsizgus marmoreus*, *Pleurotus astreatus*, *P. seesteranus*. ^L^ *Auricularia* spp.: *Auricularia auricula*, *A. cornea*, *A. cornea* (white variety).

**Table 2:** Means and standard errors (*m* ± *SE*) for developmental durations of *Bradysia impatiens* in its four life stages at eight rearing temperatures, as well as survival rates of immature insects to new filial adults.

Temperature (*T*, °C, *m* ± *s*)	Egg (d)	Larva (d)	Pupa (d)	Immature (d)	Survival Rate of Immatures (%)
*F* (*df* = 7, 276)	745.0 **	144.6 **	896.1 **	300.5 **	-
10.0 ± 0.5	14.1 ± 0.13 a A	74.8 ± 3.0 a A	21.4 ± 0.4 a A	110.3 ± 2.7 a A	14.3 (8/56) ^†^
11.6 ± 0.4	10.9 ± 0.25 b B	49.3 ± 3.1 b B	15.7 ± 0.3 b B	75.8 ± 3.2 b B	41.9 (36/86)
15.6 ± 0.1	7.1 ± 0.04 c C	24.6 ± 1.1 c C	8.8 ± 0.2 c C	40.5 ± 1.2 c C	40.7 (35/86)
18.5 ± 0.1	6.0 ± 0.06 d D	20.5 ± 0.6 d CD	6.5 ± 0.2 d D	33.0 ± 0.7 d D	50.0 (43/86)
21.2 ± 0.1	4.1 ± 0.09 e E	15.8 ± 0.7 e DE	5.0 ± 0.1 e E	24.9 ± 0.7 e E	58.1 (50/86)
24.5 ± 0.1	3.9 ± 0.09 e E	13.2 ± 0.5 ef E	4.1 ± 0.1 f F	21.1 ± 0.5 ef EF	52.3 (45/86)
27.2 ± 0.1	3.2 ± 0.04 f F	11.3 ± 0.5 f E	3.8 ± 0.1 f F	18.2 ± 0.5 f F	58.1 (50/86)
30.0 ± 0.2	2.4 ± 0.05 g G	12.2 ± 0.3 ef E	3.7 ± 0.2 f F	18.3 ± 0.3 f F	42.5 (17/40)

*F*, statistical *F*-test ratios from ANOVA among developmental durations. **, significant at *p* < 0.01, *F*-test. Means within the same column followed by the same letter in small case are not significantly different at *p* < 0.05. Means within the same column followed by the same capital letter are not significantly different at *p* < 0.01. Duncan’s multiple range test (the same below). ^†^, the figures in brackets are the numbers of new filial adults/the numbers of eggs inoculated.

**Table 3:** Linear correlation coefficients (*r*) between rearing temperatures (*T*) and developmental rates (*V*) of *Bradysia impatiens* in each life stage, threshold temperatures (*C*) and thermal constants (*K*) with their standard errors (*s_C_* and *s_K_*), and the results of the chi-square goodness-of-fit test for the newly constructed real reciprocal models [49].

Life Stage	Sample Size (*n*)	Linear Correlation Coefficient Between *T* and *V* (*r*)	Threshold Temperature (°C)		Thermal Constant (d°C)		Chi-Square Test Between *NE* and *NO* ^†^	
			*a* or *C*	*s_a_* or *s_C_*	*b* or *K*	*s_b_* or *s_K_*	*χ* ^2^	*p≥*
Egg	7	0.9904 ***	5.5	0.90	69.9	4.37	0.285	0.9996
Larva	7	0.9986 ***	6.8	0.31	230.1	5.39	0.213	0.9998
Pupa	7	0.9971 ***	6.7	0.45	75.1	2.54	0.134	1.0000
Immature	7	0.9988 ***	6.6	0.29	374.9	8.15	0.139	0.9999

***, the linear correlation coefficient (*r*) is significant at *p* < 0.001. ^†^, the symbol *NO*, standing for the average number observed, is designated to the actual mean for developmental durations of the insect in a life stage; and the symbol *NE* is the average number estimated by corresponding reciprocal model (see the text for detail).

**Table 4:** Linear correlation coefficients (*r*) between *R* and *T*, results of linear regression analysis derived by fitting the equation *R* = *a* + *bT* to the same seven data points via Method 2, and *Tth* and *K* calculated via −*a*/*b* and 1/*b* (refer to text for the meanings of *R*, *T*, *Tth* and *K*, respectively).

Life Stage	Sample Size (*n*)	Linear Correlation Coefficient Between *R* and *T* (*r*)	Linear Regression Coefficient		Parameters Calculated	
			*a*	*b*	*Tth* (°C)	*K* (d°C)
Egg	7	0.9904 ***	−0.0740	0.0140	5.3	71.3
Larva	7	0.9986 ***	−0.0295	0.0043	6.8	230.7
Pupa	7	0.9971 ***	−0.0881	0.0132	6.7	75.5
Immature	7	0.9988 ***	−0.0175	0.0027	6.6	375.8

***, the linear correlation coefficient (*r*) is significant at *p* < 0.001, the same as in Table 3.

**Table 5:** Developmental durations in immature stage (DDIS), survival rates of the immature insects (SRI), and fecundities of *Bradysia impatiens* [27] or *B*. *difformis* [22,28,34] when larvae were fed with different hosts at 25 ± 1 °C.

Host Plant or Edible Fungus	DDIS (d)	SRI (%)	Fecundity (Eggs per Female)	Reference
Chinese chive, *Allium tuberosum*	16.4 d	68.8	92.0 a	[22]
Spring onion, *Allium fistulosum*	21.1 b	57.5	85.5 a	
Garlic bulb, *Allium sativum*	22.9 a	12.5	73.9 ab	
Garlic stem, *Allium sativum*	17.6 c	30.0	73.0 ab	
Lily, *Lilium davidii* var. *unicolor*	22.8 a	31.2	52.1 b	
Oyster mushroom, *Pleurotus ostreatus*	16.8 c	79.5	66.8 a	[27]
Hime matsutake, *Agaricus subrufescens*	17.2 c	74.4	60.6 ab	
Tea tree mushroom, *Agrocybe cylindracea*	20.1 b	51.3	40.3 b	
Shiitake, *Lentinus edodes*	23.0 a	47.4	39.6 b	
Chinese chive, *Allium tuberosum*	21.4 b	72.1	91.6 a	[28]
Broad bean, *Vicia faba*	19.3 c	52.6	78.1 b	
Lettuce, *Lactuca sativa* var. *ramose*	20.0 c	45.3	64.1 c	
Chinese cabbage, *Brassica oleracea*	23.1 a	52.6	63.7 c	
Wild cabbage, *Br. oleracea* var. *rubra*	23.4 a	52.6	60.7 c	
Oyster mushroom, *Pleurotus ostreatus*	21.2 c	32.3 ab	106.4 a	[34]
Shiitake, *Lentinus edodes*	21.7 c	37.8 a	98.1 a	
Chinese chive, *Allium tuberosum*	22.8 b	32.2 ab	79.4 b	
Chinese yam, *Dioscorea opposita*	23.9 a	32.0 ab	76.5 b	
Enoki mushroom, *Flammulina velutipes*	22.6 b	20.4 b	66.7 c	
Potato, *Solanum tuberosum*	24.3 a	13.8 c	42.6 d	
Mean	21.1	44.8	70.6	

Means from the same reference (article) within the same column followed by the same letter are not significantly different at *p* < 0.05, Duncan’s multiple range test. Means are rearranged and rounded to one or two decimals, with their *SD* or *SE* omitted, but letters denoting their significance follow. The RH and photoperiod in the original references are omitted herein because of secondary factors.

## Data Availability

Most data that were analyzed and modeled during the studies are included in this published article. The rest are available from the corresponding authors on reasonable request.
